# A Relative Variation-Based Method to Unraveling Gene Regulatory Networks

**DOI:** 10.1371/journal.pone.0031194

**Published:** 2012-02-20

**Authors:** Yali Wang, Tong Zhou

**Affiliations:** 1 Department of Automation, Tsinghua University, Beijing, China; 2 Tsinghua National Laboratory for Information Science and Technology (TNList), Tsinghua University, Beijing, China; Queen's University Belfast, United Kingdom

## Abstract

Gene regulatory network (GRN) reconstruction is essential in understanding the functioning and pathology of a biological system. Extensive models and algorithms have been developed to unravel a GRN. The DREAM project aims to clarify both advantages and disadvantages of these methods from an application viewpoint. An interesting yet surprising observation is that compared with complicated methods like those based on nonlinear differential equations, etc., methods based on a simple statistics, such as the so-called 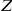-score, usually perform better. A fundamental problem with the 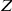-score, however, is that direct and indirect regulations can not be easily distinguished. To overcome this drawback, a relative expression level variation (RELV) based GRN inference algorithm is suggested in this paper, which consists of three major steps. Firstly, on the basis of wild type and single gene knockout/knockdown experimental data, the magnitude of RELV of a gene is estimated. Secondly, probability for the existence of a direct regulation from a perturbed gene to a measured gene is estimated, which is further utilized to estimate whether a gene can be regulated by other genes. Finally, the normalized RELVs are modified to make genes with an estimated zero in-degree have smaller RELVs in magnitude than the other genes, which is used afterwards in queuing possibilities of the existence of direct regulations among genes and therefore leads to an estimate on the GRN topology. This method can in principle avoid the so-called cascade errors under certain situations. Computational results with the Size 100 sub-challenges of DREAM3 and DREAM4 show that, compared with the 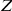-score based method, prediction performances can be substantially improved, especially the AUPR specification. Moreover, it can even outperform the best team of both DREAM3 and DREAM4. Furthermore, the high precision of the obtained most reliable predictions shows that the suggested algorithm may be very helpful in guiding biological experiment designs.

## Introduction

In the post-genomic era, one of the fundamental tasks is reconstructing gene regulatory networks (GRN) from experimental data and other a priori information. It is hoped that this reconstruction is helpful in both understanding cell functions and gaining additional insights about the processes of some complicated diseases that might lead to new target gene discovery. Recently, with the development of high-throughput technologies, such as DNA microarrays and mass spectroscopy, etc., it becomes possible to simultaneously collect thousands of gene expression data [1], [2]. Stimulated by these technology advancements, a variety of different models and methods have been proposed for GRN reconstruction, such as Boolean networks [3], [4], Bayesian networks [5], [6], information theory based algorithms [7]–[10], ordinary differential equation (ODE) based methods [11]–[13], etc. In addition, some software packages, such as GeneNet, minet, etc., have been developed [8], [9].

A challenge common to all these reverse-engineering methods is that in comparison with the dimension and complexity of a GRN, the collected experimental data is generally with a low SNR (signal-to-noise ratio) and the number of observations is not very large in every experiment. Another challenge is to evaluate the appropriateness of the assumptions adopted by these methods. To settle these problems, the Dialogue for Reverse Engineering Assessments and Methods (DREAM) project recently provided a set of benchmark networks that can be used to compare both advantages and disadvantages of different GRN topology inference methods [14]–[17]. Compared with other benchmark networks, one of the most attractive characteristics of the networks provided by the DREAM project is that they are extracted from actual biological networks and are able to represent some most important and typical biological modules. By far, it has become one of the most widely used benchmarks for GRN topology inference.

Several methods have been shown to be effective in inferring the structure of a GRN through participating the DREAM project. For example, the best performer of the DREAM3 subchallenges took an approach that firstly learns some Gaussian noise models from knockout experimental data, and then combines these results with those obtained through fitting time series experimental data to an ODE model [18]. The second place team of the DREAM3 Size 100 subchallenges utilized a mutual information (MI) based method and a so-called Inferelator 1.0 method, which takes sparsity of a GRN into account through penalizing the 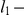
norm of the kinetic parameter vector of the ODE model [19]. On the other hand, the DREAM project organizers applied the so-called 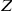-score to measure possibilities of the existence of a direct regulation from one gene to another gene [16]. Surprisingly but also interestingly, this simple method was proved to be placed at respectively the first (tie) and the third for the Size 100 subchallenges of DREAM3 and DREAM4.

From a statistical point of view, the 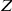-score based method is actually a 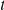-test [20]. More precisely, to determine whether gene 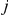 has a direct regulation on gene 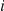, it utilizes the absolute expression level variation (AELV) of gene 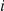 from the wild type after a perturbation on gene 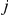. The larger the magnitude of this AELV is, the more unlikely that the change is due to measurement noise, and thus the larger the probability that gene 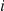 is directly regulated by gene 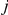. This AELV, however, is sometimes not very effective in distinguishing a direct regulation from an indirect one, as possibilities can hardly be excluded that an indirect regulation causes an AELV larger in magnitude than some direct regulations [21]. To reduce estimation errors caused by indirect regulations, which is often called cascade errors [21], [22], the best performer of the Size 100 subchallenges of DREAM4 suggested to refine the results of the 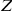-score based method through down ranking some feedforward edges [22]. Basically, the idea behind this treatment is to remove every direct regulation between two different genes in a GRN estimate, provided that it does not belong to a cycle and there exists another direct or indirect regulation between these two genes. This procedure has significantly improved the adopted estimation specifications, and therefore shown its efficiency in GRN topology estimations [22].

The results of [22] are encouraging. It seems, however, that further efforts are still required to make the estimation procedure applicable to practical problems, noting that as reported in [22], its prediction accuracy for some networks is still not very high, and thresholds exist that are significantly different from the recommended one but are capable of leading to a much better network structure estimate. In addition to this, our computational experiences with this method show that its precision-recall (PR) curve is still not very satisfactory for some networks. A detailed discussion on this issue is provided in the subsection of Further Discussions of the section of Results and Discussions.

To achieve a better GRN structure estimation, an innovative technique is proposed in this paper for GRN topology inference. The ideas behind the developed algorithm are relatively simple. That is, rather than absolute change, relative variation of gene expression level is adopted in measuring possibilities of the existence of a direct regulation between two different genes of a GRN. This algorithm consists of three major steps. That is, magnitude estimation and normalization of the relative variations, estimation of genes not regulated by other genes, modification of the normalized estimate for the magnitude of the relative variations and GRN topology identification. In the first step, relative expression level variation (RELV) of a gene is estimated using experimental data before and after another gene of the same GRN has been perturbed. This estimate is further normalized to reflect effort differences of regulating distinct genes. In the second step, on the basis of the estimated probability that the magnitude of the RELV of a gene is greater than a prescribed value, genes with a zero in-degree are estimated. Finally, in the third step, every normalized magnitude of the AELV of a gene with an estimated nonzero in-degree is adjusted to be greater than those with an estimated zero in-degree. Computational experiences with the Size 100 network inference subchallenges of both DREAM3 and DREAM4, as well as some other simulated large scale GRNs, show that this method can significantly outperform not only the 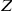-score based method, but also the best teams who utilized an integration of several widely adopted methods. The suggested method has also been integrated with the so-called down ranking method, which is recommended by the best network inference team of DREAM4. Once again, it has been confirmed through actual computations that this method is helpful in reducing cascade errors. The corresponding improvement, however, is not as significant as that to the 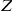-score based method. This means that some cascade errors have been reduced by the suggested method, which confirms from another aspect that the suggested method is really effective in distinguishing direct and indirect regulations of a GRN.

The outline of this paper is as follows. At first, the relative variation based estimation algorithm is illustrated. A technique is also provided that can integrate GRN topology prediction results using respectively steady state knockdown and knockout experimental data, as well as a procedure that integrates the method suggested in this paper with the so-called down ranking method. Afterwards, the proposed estimation method is assessed using the data sets of the Size 100 subchallenges of both DREAM3 and DREAM4. Variations of estimation performances with respect to parameters of the suggested method have also been reported, as well as estimation results using both steady state knockdown and knockout experimental data. In addition, estimation results are also given in which the suggested method is integrated with the so-called down ranking method. Finally, some concluding remarks are given about characteristics of the suggested method, as well as some future works worthy of further efforts.

## Materials and Methods

Concerning a GRN with 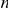 genes, assume that measurement errors affect experimental data in an additive way, as well as that measurement errors with the expression level of gene 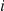 have an independent and identical normal distribution 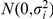. Let 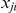 and 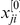 represent respectively the observed and the actual gene expression levels of gene 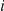 when gene 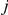 is knocked out or knocked down, and 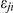 the corresponding measurement error. Then, it is obvious from these representations that
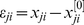
(1)Moreover, denote by 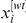 and 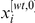 respectively the observed and the actual expression levels of gene 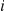 in the wild type, and 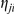 the steady expression level variation of gene 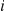 after the knockout/knockdown of gene 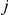. Then, from its definition, we have that 
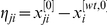
, and from this relation, straightforward algebraic operations show that
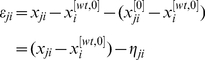
(2)


Define the RELV (relative expression level variation) of gene 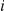 resulted from a perturbation on gene 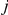, denote it by 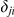, as
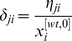
(3)Note that in a GRN, every gene can usually be approximately assumed to be in one of the following two states, expressed and unexpressed states. Moreover, expression levels, that is, concentrations of the corresponding proteins or mRNAs, etc., of distinct genes usually take very different values, and sometimes these values may even have different orders [1], [11], [23]. These imply that when a gene is knocked out or knocked down, absolute variations of the expression levels of genes regulated by this externally perturbed gene may have very different magnitudes. These characteristics of 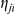 are not very attractive in GRN topology estimation, as they imply that the magnitude of 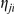 due to an indirect regulation may sometimes be significantly larger than that due to a direct regulation. On the other hand, when the RELV is utilized, the aforementioned problems can be partly overcome. More specifically, RELVs of every gene are roughly of the same order, which makes their comparisons more biologically significant than the AELV that is important in GRN structure estimations. In addition, if in a pathway of GRNs, every direct regulation, say, that from gene 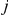 to gene 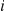, make a relative variation of the concentrations of the proteins or mRNAs, etc., related to the regulated gene 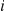 at most as large as that of the regulation gene 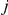, then, it is obvious that in this pathway, the magnitude of every RELV due to an indirect regulation is certainly not greater than that due to a direct regulation. From these considerations, it appears that RELV is more attractive than AELV in GRN topology estimations.

But it is worthwhile to emphasize that self-activation usually exists in GRNs [23], [24], which may make the RELVs of a pathway be amplified during cascade gene connections. This means that the aforementioned assumption may not be satisfied by every pathway of a GRN. To make things worse, for some genes of a GRN, there exist more than 1 directed pathways from one gene to another gene [14], [24]. For example, when gene 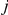 directly regulates gene 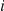 (through its proteins), it is possible that gene 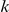 is also directly regulated by gene 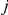 and gene 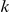 directly regulates gene 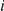 further. Under such a situation, when gene 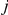 is externally perturbed, the RELV of gene 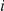 is due to both direct and indirect regulations. If one of these two regulations has an activation effect and the other has a suppression effect, then, their composite effects may significantly weaken that of the direct regulation and therefore result in an incorrect estimate using the above assumption, noting that generally, the RELV of a gene should be estimated from experimental data.

The above arguments show that although the RELV of a gene has some attractive properties in GRN topology estimations, it is still not very clear whether or not the adopted assumption is reasonable for most pathways of a GRN from a biological viewpoint. It appears, however, from our computational experiences, some of which are reported in the section of Results and Discussions, that this assumption may have some nice biological interpretations and is satisfied by many regulations existent in a GRN.

These arguments mean that relative concentration variation is, at least under many situations, able to differentiate direct and indirect regulations of a GRN, and is therefore more effective than the absolute one in GRN topology estimations, noting that indirect regulations are rich in a GRN. These arguments also imply that the larger the magnitude of 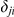 is, the more unlikely that the expression level variation of gene 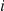 after perturbing gene 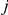 is due to indirect regulations, and thus the larger the probability that gene 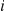 is directly regulated by gene 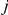.

### RELV Estimation

As 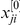 is generally not available from experiments, an estimate for 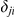 should be used in GRN topology inference. To obtain this estimate, the set to which 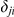 belongs most likely with a fixed probability is considered. Recalling that 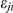 is assumed to have a normal distribution 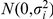, this is equivalent to compute the minimal interval that contains an estimate of 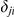 when the measurement noise 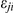 is assumed to be not greater in magnitude than 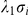 for a fixed non-negative 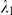. On the basis of this observation and Equation (2), as well as the fact that both 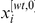 and 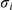 are always positive, it can be directly shown that
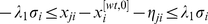
(4)

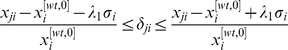
(5)Therefore,
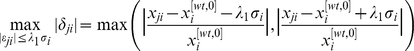


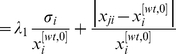
(6)


Note that in GRN topology inference, the sequence of probability of the existence of a direct regulation from one gene to another gene plays the most essential role. Moreover, it has been argued that the larger the absolute value of 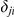, the higher the probability that gene 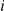 is directly regulated by gene 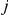. Based on these considerations, the element with the maximal magnitude of the 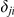
s satisfying Equation (5) is taken as its estimate. Denote this estimate by 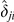. Then, from Equation (6), we have that
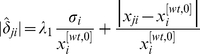
(7)and its value can be calculated from experimental data, provided that both 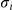 and 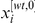 are known.

In practical applications, however, these two parameters are generally not available and should also be estimated from experimental data. If the set of genes that do not affect gene 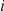, denote it by 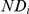, is known, then, some widely adopted estimates for 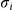 and 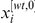 are respectively as
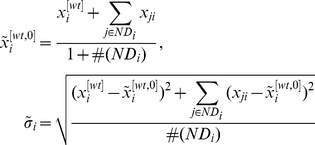
(8)in which 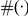 stands for the element number of a set [25], [26]. However, the set 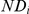 is usually unknown before GRN topology inference, which invalidates adoption of the above estimates. On the other hand, it is now widely recognized that a large scale GRN usually has a sparse topology [11], [13], [22], which means that for most genes of a large scale GRN, 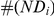 is very close to 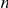 which stands for the number of its genes. This means that in estimating 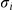 and 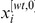, essential differences will not arise for most genes even if 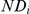 is taken to be the whole set of the genes of a GRN. Based on these considerations, the following estimates are adopted in this paper for 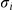 and 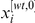, in which differences have not been taken into account between a measurement for the expression level of gene 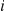 in the wild-type and those when some other genes have been knocked out and/or knocked down.
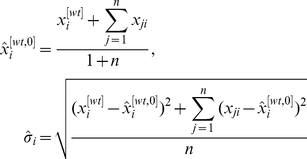
(9)Although these estimates may be crude, they are widely adopted in GRN topology estimation and are capable of leading to a good network estimate [16], [18]. For example, both the best team of DREAM3 and that of DREAM4 have taken these values as estimates for 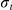 and 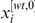 [19], [22].

When estimates for 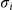 and 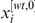 are available, an estimate for 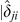 of Equation (7) can be obtained from experimental data 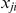 through replacing both 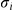 and 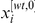 by their estimates respectively. Denote this estimate by 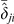. Then, we have that
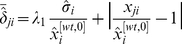
(10)


### RELV Normalization

Define a 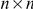 dimensional matrix 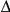 with its 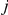-th row 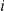-th column element being the estimate of 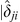 when 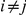 and its diagonal element being zero, and denote its 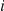-th column vector by 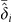. Then, the above derivations make it clear that this matrix contains information about the probability of the existence of a direct regulation between any two different genes in a GRN. However, to infer the structure of a GRN from the aforementioned matrix 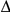, an important fact must be taken into account. That is, in a GRN, some genes may be easily regulated by other genes, while regulations on some other genes may need more efforts [23], [24]. As a matter of fact, even when every direct regulation of a pathway in a GRN satisfies that RELV of the regulated gene is not greater than that of a regulation gene, it is still possible that direct regulations to different genes lead to different magnitudes of these variations of the regulated genes, although under such a situation, a direct regulation of this pathway certainly makes the RELV of the regulated gene have a magnitude not smaller than that of every indirect one. Therefore, in order to obtain a good estimate from the matrix 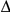 about the topology of a GRN, an appropriate normalization is still required for the estimated 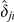
s among different genes.

Although it is still not very clear how to make a biologically significant normalization among the RELVs of different genes, as a primary study, it is suggested in this paper to use the 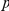-norm of the vector 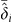 and the geometric average of its non-zero elements to achieve this objective, which are widely adopted in many fields like system analysis and synthesis, signal processing, etc., and have shown their efficacy [13], [25], [27]. While their effectiveness in GRN topology estimation is still not very clear theoretically, our computational experiences, part of them are given in the section of Results and Discussions of this paper, show that they are able to lead to an estimate much better than that without normalizations. More specifically, when the 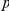-norm and the geometric average are respectively used in this normalization, the 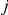-th row 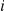-th column element of the matrix 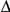, that is, 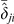, is respectively replaced by
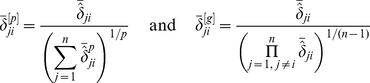
(11)It is worthwhile to note that this normalization does not change the diagonal elements, which is important as self regulation can hardly be identified from either knockout or knockdown experimental data. For presentation conciseness, the normalized matrix 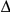 using the vector 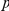-norm and the geometric average is denoted respectively by 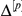 and 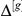 in the rest of this paper.

### Estimation of Genes with a Zero In-degree

While normalization is helpful in balancing RELVs among different genes, another problem arises in GRN topology estimations. That is, this normalization usually leads to wrong estimates in network inference for genes not regulated by other genes. This is because that although for these genes, the corresponding computed 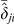
s are generally very small, some of their normalized values may have a comparable magnitude with those of a gene that is regulated by other genes. Note that in a GRN, nodes with a zero in-degree, that is, genes that can not be regulated by other genes, extensively exist [16], [23]. Therefore, special cautions must be taken to deal with them in inferring the structure of a GRN.

To distinguish genes that can be and can not be regulated by other genes, once again RELVs of a gene are considered when another gene is knocked out and/or knocked down, but in a different way. It is worthwhile to point that in principle, it is also possible to use 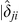 in estimating genes that are with a zero in-degree. However, actual computations show that the corresponding estimate is not as effective as the following estimate. More precisely, for a prescribed 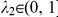, it is reasonable to regard that there exists a direct regulation from gene 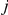 to gene 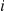 when 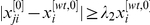. Otherwise, gene 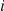 is considered not to be directly regulated by gene 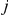. As 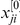 can hardly be estimated with an acceptable accuracy from experimental data in actual GRN structure identification, probability is taken as a measure for the existence of a direct regulation from gene 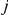 to gene 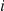. Recall that measurement errors are assumed to affect experimental data additively. From Equation (2) and the definition of 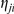, it is obvious that 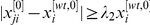 is equivalent to
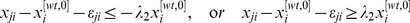
(12)which can be further expressed as
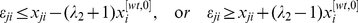
(13)


On the other hand, note that 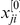 stands for the actual expression level of gene 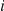 when gene 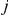 is externally perturbed, and therefore can not take a negative value. It is straightforward from this fact and Equation (1) that the following inequality should always be satisfied by the measurement error 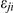.
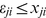
(14)


Summarizing Equations (13) and (14), it can be declared that to guarantee the existence of a direct regulation from gene 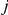 to gene 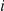, it is necessary and sufficient that
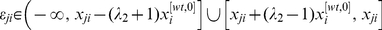
(15)Noting that measurement errors are also assumed to have a normal distribution 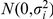, the above equation makes the probability computable for the existence of a direct regulation from gene 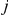 to gene 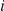, provided that both 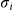 and 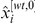 are available. In practical inference, 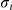 and 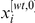 are usually replaced by their estimates 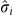 and 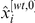 that are provided in Equation (9).

Denote the corresponding calculated probability by 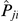. Then, the above arguments make it clear that
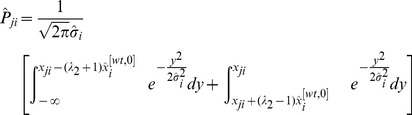
(16)Moreover, the larger the 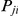 is, the higher the confidence is that gene 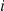 is directly regulated by gene 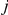. Let 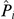 represent the maximum value of 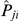 when 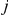 varies over all the integers between 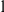 and 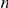 except 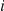, that is, 
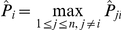
. The above arguments imply that if 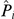 takes a small value, then, it is very possible that gene 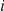 is not regulated by any other genes of the GRN. In other words, the in-degree of this gene is equal to zero with a high probability.

To estimate genes that has a zero in-degree, both absolute value and relative largeness of 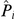 are considered. This can make all the genes with an estimated zero in-degree have an estimate of the probability of being regulated by other genes not only very small, but also significantly smaller than that of every gene with an estimated nonzero in degree. More precisely, rearrange 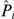 in an increasing order, that is, 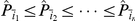 in which 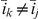 when 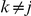 and 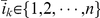. Define 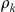 as 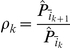, 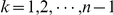. Let 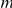 denote the first integer with which 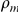 takes the greatest value under the condition that 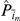 belongs to 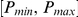 for some prescribed 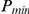 and 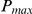, then, all genes numbered as 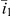, 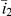, 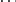, and 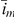 are regarded not to be regulated by any other genes of the GRN.

### RELV Magnitude Modification

With the above estimate about genes of a GRN that have a zero in-degree, the normalized RELV matrices 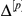 and 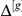 are modified, in order to get a better estimate about its structure. As genes estimated to be of a zero in-degree generally has a low probability of being regulated by other genes, its corresponding normalized RELVs must be adjusted to have a lower rank than those of genes that might be regulated by other genes. To achieve this objective, define 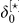 as the maximal magnitude of the normalized RELVs of the genes estimated to be not regulated by any other genes. That is,
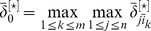
(17)in which 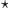 can be either 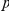 or 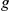. With this value, the normalized RELVs are modified as follows,
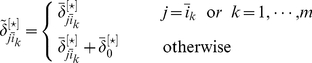
(18)This modification makes every RELV of a gene possibly regulated by other genes greater in magnitude than any RELV of a gene estimated to be of a zero in-degree.

Denote by 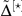 the 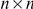 dimensional matrix with its 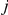-th row 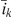-th column element being 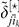, 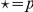 or 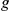. Elements of this matrix are directly used to infer the structure of a GRN, according to the principle that the bigger the 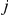-th row 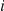-th element is, the higher the probability is that gene 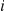 is directly regulated by gene 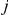.

### Estimation Algorithm

In summary, to estimate the structure of a GRN, it is assumed in this paper that measurement errors in the expression levels of every gene have an independent and identical normal distribution, and affect experimental data additively. On the basis of the concept of the RELV of a gene, an algorithm is suggested in this paper for identifying direct regulations of a GRN. This algorithm consists of the following three main steps.

1. Using a prescribed 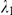 and the estimates for 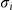 (the standard variance of measurement errors) and 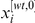 (the wild type expression level of gene 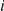), which are given in Equation (9), as well as Equations (10) and (11), calculate the matrices 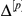 or 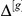 consisting of the normalized magnitudes of the estimates of the RELVs for every gene in a GRN. This is equivalent to construct the matrix 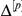 or the matrix 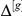 respectively as 
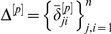
 or 
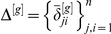
.

2. On the basis of Equations (9) and (16), as well as a prescribed 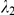, calculate the estimate for the probability that gene 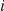 is directly regulated by gene 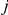, that is, 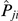. Compute 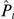 as 
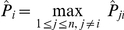
. Rearrange 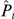 into a monotonically increasing sequence 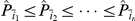. Using some prescribed thresholds for the minimum and maximum of 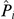, say, 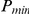 and 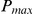, determine the gene 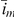 that has a 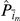 belonging to 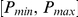 and makes 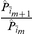 reach its maximum in the first time. Designate the in-degree of genes 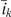, 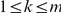, to be zero.

3. Modify the matrices 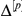 or 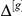 according to Equations (17) and (18). Using elements of these modified matrices, queue possibilities of the existence of a direct regulation from the gene with the same number of the row to the gene with the same number of the column. The bigger this element is, the higher the confidence is for the existence.

### Integration of Knockout and Knockdown Data

Currently, biological experiments can provide both steady state data and time series data. In addition, an experiment can be performed through knocking out a single gene, knocking down a single gene or simultaneously perturbing several genes. In this subsection, a method is proposed to integrate estimation results obtained respectively from steady state knockout and knockdown experimental data. Rather than to develop an efficient integration method, the purposes of this investigation are mainly to clarify characteristics of these types of experimental data in GRN topology estimations with the method suggested in this paper.

The integration method suggested in this paper is similar to cross validations [25], [26]. That is, if a consistent estimate is obtained from different experimental data, then, confidence is strengthened about the correctness of this estimate. More specifically, if from both the knockout and the knockdown experimental data, the estimated magnitude of the RELV corresponding to a possible direct regulation has a large value, then, confidence about the existence of this direct regulation is increased. As a large scale GRN usually has a sparse topology, it appears reasonable to only modify a few RELVs for every gene in this integration. Moreover, as knockout experimental data is widely believed to be more informative than knockdown experimental data in GRN topology identification, for example, observations from the reported results of the DREAM project show that estimation performances with knockdown experimental data are usually significantly worse than those with knockout experimental data [16], [22], a higher confidence is given to the knockout experimental data based estimates.

In this paper, it is suggested to modify the first 5 biggest RELVs of a gene obtained from knockout experimental data, provided that this gene is estimated to have a nonzero in-degree. More precisely, let 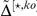 and 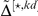 denote the 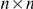 dimensional matrices consisting respectively of the modified normalized RELVs obtained from knockout and knockdown experimental data, 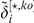 and 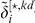 their 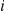-th column vectors, and 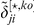 and 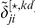 their 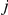-th row 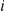-th column elements. Here, 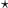 can be either 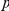 or 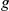, and 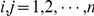. If a gene, say, gene 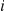, is estimated to have a nonzero in-degree using the knockout experimental data, which is equivalent to 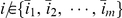, then, its modified normalized RELVs will be further adjusted according to the following procedures.

- For any 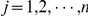, if 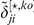 belongs to the first 5 biggest elements of the vector 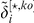, and 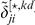 is among the first 3 biggest elements of the vector 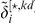, increase 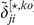 to 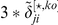.
- For any 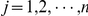, if 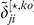 belongs to the first 5 biggest elements of the vector 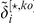, and 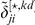 is among the 4th to 8th biggest elements of the vector 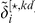, increase 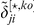 to 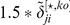.
- For any 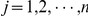, if 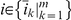, or 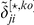 does not belong to the first 5 biggest elements of the vector 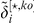, or 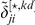 is not among the first 8 biggest elements of the vector 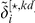, keep 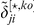 unchanged.

Denote the adjusted 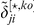 by 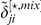. Then, topology estimation for a GRN can be performed on the basis of 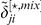 according to the same manner as that using 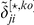.

### Integration with the Down Ranking Method

In reducing false positive errors in GRN topology inference, the so-called down ranking method has been proved to be very effective [22]. While the objectives of this method are almost the same as those of the algorithm suggested in this paper, different approaches have been utilized. Briefly, in the down ranking algorithm, it is assumed that an a priori estimation about the topology of a GRN has been obtained by some methods, and if a direct path between two genes is not in a cycle and there is another direct or indirect path between these two genes in the estimated GRN structure, then, the former direct path should be deleted. This idea has been further extended to the so-called strongly connected components. A detailed description can be found in [22].

In this subsection, a procedure is suggested to integrate the algorithm suggested in this paper with this down ranking method. The major proposes are to see whether performances in GRN topology estimation can be further improved, as well as whether the cascade errors reduced by the down ranking method can also be reduced by the method suggested in this paper. Taking into account characteristics of these two different methods, they are integrated in the following way, in which 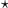 can be either 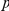 or 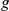.

1. Compute elements of the matrix 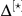 using Equation (11) and knockout experimental data.
2. For a given threshold value, say, 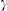, a matrix 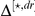 is obtained from the previously obtained matrix 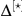, using the down ranking algorithm.
3. For every 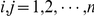 with 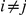, compute the estimate 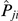 using Equation (16) that stands for the probability of the existence of a direct regulation on gene 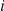 from gene 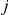. Similar to that of the 2nd step of the estimation algorithm, estimate genes with a zero in-degree using these probabilities and some prescribed 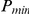 and 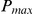. Modify the matrix 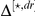 by the same token as that of the matrix 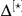, on the basis of (17) and (18). Denote the modified matrix by 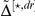.
4. Queue possibilities of the existence of a direct regulations in the GRN according to the elements of the matrix 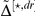, in the same way as that without method integrations in which the matrix 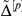 or 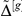 is used.

Note that in the estimation algorithm suggested in this paper, the matrix 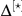, 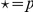 or 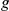, has been normalized which makes every element of this matrix belong to 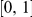. This implies that when the down ranking method is applied to the matrix 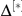, a meaningful threshold value 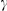 should also belong to this interval. This is different from the situation when the 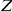-score based method is integrated with, in which the computed 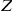-scores between two different genes may vary in a much larger interval, that leads to a much bigger set for searching the optimal threshold value 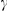.

## Results and Discussion

### Data Sets and Assessment Metrics

To illustrate the effectiveness of the developed inference algorithm, tests are performed on the Size 100 Network subchallenges of both DREAM3 and DREAM4, using the data set provided by the organizers. These subchallenges are designed to assess performances of an identification method for the structure of a large scale GRN [16]. They respectively contain 5 different benchmark networks which were obtained through extracting some important and typical modules from actual biological networks. There are three types of experimental data for each subchallenge, which are respectively knockout experimental data, knockdown experimental data, and time series experimental data. Predictions are compared with the actual structure of the networks by the DREAM project organizers using the following two different metrics in topology prediction accuracy evaluations.

- AUPR: The area under the PR (precision-recall) curve;
- AUROC: The area under the receiver operating characteristic (ROC) curve.

Moreover, for every network of DREAM3 and DREAM4, the 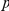-values of the AUPR and AUROC specifications, which indicate the probability that random predictions would have the same or better performances, are computed, and finally a score is calculated using these 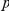-values. More specifically, the logarithm of the geometric mean is calculated respectively for both the 5 AUROC 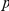-values and the 5 AUPR 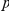-values, and the score is taken as the absolute value of the average of these two logarithms. More detailed explanations can be found in [15], [16] or the web site of the DREAM project at http://wiki.c2b2.columbia.edu/dream/. A larger score indicates a better performance of the adopted inference algorithm.

Noting that the GRN inference algorithms developed in the previous section are applicable only to steady state experimental data, concentrations of this section are focused on knockout and knockdown experimental data. As the suggested estimation algorithm without either data integration or method integration consistently gives much better performances when the knockout experimental data are used for the Size 100 subchallenges of both DREAM3 and DREAM4, which is in a good agreement with other methods reported by the participants of the DREAM project [16], [19], [22], the corresponding results are at first reported.

### Performances for the Knockout Data

Using the knockout experimental data provided by the DREAM project organizer, GRN topology inference is performed for the Size 100 subchallenges of both DREAM3 and DREAM4. To investigate influences of different normalization on the prediction accuracy of the estimation algorithm, 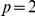 is firstly adopted for the 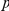-norm based normalization, which is widely utilized in fields like system analysis and synthesis, signal processing, etc.[13], [25], [27]. Moreover, 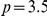 is also utilized which is found to be close to the optimal one for most networks of DREAM3 and DREAM4. In addition, the optimal 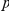 is also searched for the 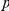-norm based normalization over the interval 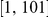 for the Net3 network of DREAM4, and 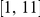 for all the other networks, through an equally spaced sampling with 100 samples. This is because that actual computations show that for the Net3 network, the AUPR specification does not take its optimal value when the parameter 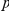 is restricted to the interval 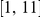. In fact, it still increases around 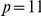. In this optimization, the desirable 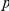 is selected to be the sample that maximizes the AUPR specification. This is mainly because that due to some precision problems of the score computation method provided by the organizers, the computed 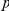-value of some networks become zero which makes it impossible to compute the score of the corresponding estimation algorithm. These problems can also be understood from the results reported in Table 1, in which several computed 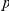-values are zero. On the other hand, according to our computational experiences, significant improvement on the AUROC specification appears much more difficult. The results are provided in Tables 1 and 2, in which 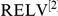, 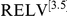, 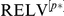 represent respectively the results for the algorithm using the 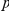-norm based normalization with 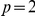, 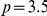 and the optimal 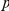; while 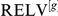 those for the algorithm using the geometric average based normalization. With a little abuse of terminology, in the rest of this paper, these representations are used to indicate the suggested estimation method with the corresponding normalization, in order to avoid awkward statements.

**Table 1:** Prediction Performances for the DREAM3 Networks.†

			Ecoli1	Ecoli2	Yeast1	Yeast2	Yeast3	ARPV
AUROC	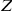 -Score	Area	0.9131	0.9633	0.8869	0.8470	0.7882	
		p-value	1.7020 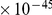	2.1928 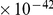	4.1185 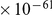	2.9808 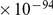	7.7889 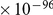	
	Best Team	Area	**0.948**	0.960	**0.915**	0.856	0.783	
		p-value	1.226 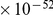	5.876 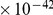	4.087 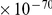	5.755 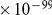	1.722 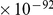	
	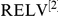	Area	0.9243	0.9662	0.8997	0.8565	0.7971	
		p-value	9.6983 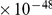	7.8920 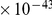	4.3987 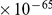	4.7112 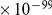	1.7888 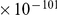	
		RPV-Z	1.2377%	0.3010%	1.4432%	1.1216%	1.1292%	1.0465%
		RPV-B	−2.5000%	0.6458%	−1.6721%	0.0584%	1.8008%	−0.3334%
	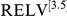	Area	0.9262	0.9632	0.9011	**0.8584**	**0.7999**	
		p-value	3.9881 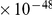	2.2713 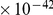	1.5888 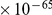	5.0037 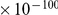	2.7921 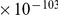	
		RPV-Z	1.4458%	−0.0104%	1.6011%	1.3459%	1.4844%	1.1734%
		RPV-B	−2.2996%	0.3333%	−1.5191%	0.2840%	2.1584%	−0.2093%
	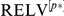	Area	*0.9252*	*0.9634*	*0.9019*	*0.8572*	*0.7998*	
		p-value	*6.3691* 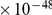	*2.1171* 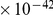	*8.8649* 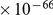	*2.0648* 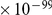	*3.2413* 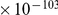	
		RPV-Z	1.3363%	0.0104%	1.6913%	1.2043%	1.4717%	1.1428%
		RPV-B	−2.4051%	0.3542%	−1.4317%	0.1402%	2.1456%	−0.2394%
		Opt. 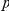	(2.7000)	(3.3000)	(4.7000)	(2.4000)	(3.3000)	
	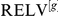	Area	0.9229	**0.9691**	0.8948	0.8508	0.7925	
		p-value	1.8625 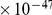	2.8270 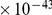	1.5099 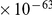	3.6937 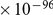	1.5372 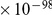	
		RPV-Z	1.0843%	0.6021%	0.8879%	0.4480%	0.5494%	0.7143%
		RPV-B	−2.6477%	0.9479%	−2.2104%	−0.6081%	1.2171%	−0.6602%
AUPR	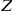 -Score	Area	0.6919	0.8536	0.5758	0.5076	0.4447	
		p-value	3.3473 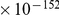	3.4060 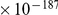	5.5895 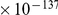	1.7370 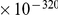	0.0000 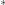	
	Best Team	Area	0.694	0.806	0.493	0.469	0.433	
		p-value	1.029 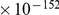	9.154 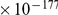	7.306 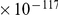	7.580 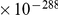	0.000*	
	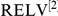	Area	0.7230	0.8674	0.6013	0.5163	0.4569	
		p-value	3.9785 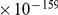	3.0047 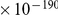	3.5106 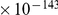	0.0000*	0.0000*	
		RPV-Z	4.4798%	1.6286%	4.4286%	1.7139%	2.7434%	2.9989%
		RPV-B	4.1787%	7.6179%	21.9675%	10.0853%	5.5196%	9.8738%
	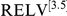	Area	0.7260	0.8686	0.6191	0.5130	0.4625	
		p-value	8.5451 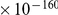	1.6300 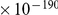	1.6449 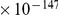	0.0000*	0.0000*	
		RPV-Z	4.9133%	1.7692%	7.5200%	1.0638%	4.0027%	3.8538%
		RPV-B	4.6110%	7.7667%	25.5781%	9.3817%	6.8129%	10.8301%
	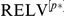	Area	***0.7293***	***0.8688***	***0.6225***	***0.5167***	***0.4628***	
		p-value	*1.5737* 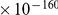	*1.4721* 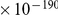	*2.4503* 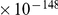	*0.0000* *	*0.0000* *	
		RPV-Z	5.3902%	1.7926%	8.1105%	1.7928%	4.0702%	4.2312%
		RPV-B	5.0865%	7.7916%	26.2677%	10.1706%	6.8822%	11.2397%
		Opt. 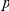	(2.7000)	(3.3000)	(4.7000)	(2.4000)	(3.3000)	
	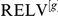	Area	0.6988	0.8539	0.5772	0.5088	0.4468	
		p-value	9.7338 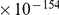	2.9231 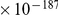	2.5519 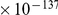	1.5200 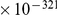	0.0000*	
		RPV-Z	0.9827%	0.0469%	0.2397%	0.2456%	0.4725%	0.3975%
		RPV-B	0.6916%	5.9429%	17.0751%	8.4961%	3.1874%	7.0786%

RPV-Z: relative performance variation with respect to the 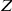-score based method; RPV-B: relative performance variation with respect to the best team; ARPV: averaged relative performance variation of the 5 networks.

†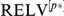, which stands for the method with the optimal normalization parameter 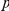, generally can not be applied in actual estimations. The purposes to include its inference results here are only to make it clear that significant estimation performance degradation does not occur when the parameter 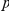 deviates from its optimal value.

*Due to some precision issues of the method suggested by the DREAM project organizers, these 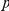-values can not be distinguished from zero in actual computations, which makes it impossible to compare scores of the adopted GRN topology estimation methods.

**Table 2:** Prediction Performances for the DREAM4 Networks.†

			Net1	Net2	Net3	Net4	Net5	ARPV	Score
AUROC	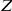 -Score	Area	0.9132	0.8015	0.8328	0.8424	0.7583		
		p-value	7.1632 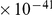	4.3251 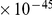	3.6020 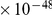	4.6477 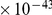	7.0347 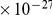		
	Best Team	Area	0.914	0.801	**0.833**	0.842	0.759		
		p-value	6.214 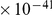	4.325 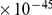	3.187 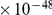	6.503 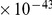	5.070 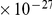		
	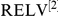	Area	0.9168	**0.8141**	0.8271	0.8498	0.7699		
		p-value	1.9857 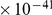	1.8773 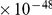	1.1463 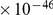	1.2941 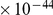	5.6222 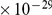		
		RPV-Z	0.3942%	1.5721%	−0.6844%	0.8784%	1.5297%	0.7380%	
		RPV-B	0.3063%	1.6355%	−0.7083%	0.9264%	1.4361%	0.7192%	
	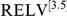	Area	0.9147	0.8123	0.8274	0.8500	**0.7711**		
		p-value	4.2006 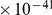	5.4649 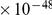	1.0162 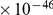	1.1739 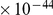	3.3778 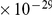		
		RPV-Z	0.1643%	1.3475%	−0.6484%	0.9022%	1.6880%	0.6907%	
		RPV-B	0.0766%	1.4107%	−0.6723%	0.9501%	1.5942%	0.6719%	
	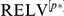	Area	*0.9147*	*0.8126*	*0.8266*	***0.8502***	*0.7710*		
		p-value	*4.2006* 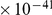	*4.5274* 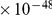	*1.5488* 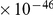	*1.0647* 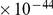	*3.5245* 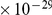		
		RPV-Z	0.1643%	1.3849%	−0.7445%	0.9259%	1.6748%	0.6811%	
		RPV-B	0.0766%	1.4482%	−0.7683%	0.9739%	1.5810%	0.6622%	
		Opt. 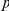	(3.5000)	(3.3000)	(55.0000)	(3.0000)	(4.4000)		
	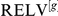	Area	**0.9192**	0.8127	0.8282	0.8451	0.7620		
		p-value	8.4095 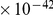	4.2520 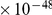	6.2716 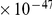	1.2663 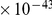	1.5363 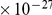		
		RPV-Z	0.6570%	1.3974%	−0.5524%	0.3205%	0.4879%	0.4621%	
		RPV-B	0.5689%	1.4607%	−0.5762%	0.3682%	0.3953%	0.4434%	
AUPR	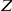 -Score	Area	0.4927	0.3881	0.3814	0.3685	0.1703		70.3408
		p-value	4.8276 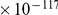	6.9794 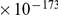	7.0623 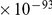	9.1672 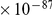	4.9150 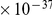		
	Best Team	Area	0.536	0.377	0.390	0.349	**0.213**		71.589
		p-value	1.197 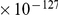	6.141 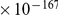	5.195 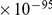	4.780 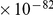	2.507 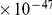		
	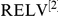	Area	0.5274	0.4011	0.3935	0.3806	0.1836		73.4399
		p-value	1.5995 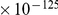	4.5994 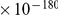	5.9685 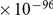	9.4155 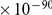	3.1017 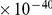		
		RPV-Z	7.0428%	3.3497%	3.1725%	3.2836%	7.8097%	4.9317%	
		RPV-B	−1.6045%	6.3926%	0.8974%	9.0544%	−13.8028%	0.1874%	
	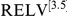	Area	**0.5638**	**0.4100**	0.4061	0.3901	0.1928		75.5441
		p-value	2.0362 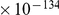	5.7745 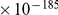	3.7653 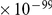	4.4903 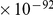	1.9708 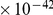		
		RPV-Z	14.4307%	5.6429%	6.4761%	5.8616%	13.2120%	9.1247%	
		RPV-B	5.1866%	8.7533%	4.1282%	11.7765%	−9.4836%	4.0722%	
	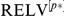	Area	***0.5638***	***0.4100***	***0.4195***	***0.3908***	*0.1967*		
		p-value	*2.0362* 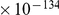	*5.7745* 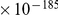	*1.4879* 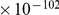	*2.8490* 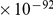	*2.3084* 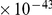		
		RPV-Z	14.4307%	5.6429%	9.9895%	6.0516%	15.5021%	10.3233%	*75.9840*
		RPV-B	5.1866%	8.7533%	7.5641%	11.9771%	−7.6526%	5.1657%	
		Opt. 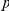	(3.5000)	(3.3000)	(55.0000)	(3.0000)	(4.4000)		
	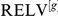	Area	0.4930	0.3904	0.3857	0.3730	0.1695		71.0752
		p-value	4.0777 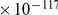	3.3976 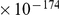	5.7129 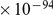	7.0934 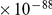	7.2225 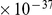		
		RPV-Z	0.0609%	0.5926%	1.1274%	1.2212%	−0.4698%	0.5065%	
		RPV-B	−8.0224%	3.5544%	−1.1026%	6.8768%	−20.4225%	−3.8233%	

RPV-Z: relative performance variation with respect to the 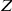-score based method; RPV-B: relative performance variation with respect to the best team; ARPV: averaged relative performance variation of the 5 networks.

† The purposes to include the inference results of 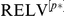 are completely the same as those of Table 1. That is, to clarify that deviation of the parameter 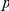 from its optimal value usually does not lead to significant estimation performance degradations.

In all these estimations, 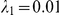, 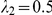 and 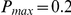 are utilized. In addition, 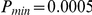 and 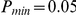 are respectively adopted for the subchallenges of DREAM3 and DREAM4. To compare prediction performances with the 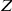-score based method and the best team, the corresponding specifications are also included in these tables. It is worthwhile to note that the estimation accuracy specifications of the best team included here are obtained directly from the web site of the DREAM project. Their digit lengthes are different from the other results that are obtained through actual computations. The best values of the AUROC and the AUPR specifications for each network are written in boldface. In addition, relative performance variation is also provided for each network, immediately below the 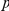-values of the estimation specifications. The first line (RPV-Z) gives results compared with the 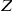-score based method, while the second line (RPV-B) those with the best team. The averaged relative performance variation (ARPV) is provided immediately after the comparisons for each network. Furthermore, the optimal 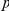 for each network is given in parentheses in the last line of the 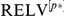 row. In the last column of Table 2, the obtained scores are also given for each method in the same row of their AUPR values. It should be pointed out that in Table 1, due to some technical issues with the software provided by the DREAM project organizers, the score can not be calculated for the best team of DREAM3 and is designated to be 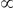 to facilitate comparisons with other methods, which is resulted from the high value of the AUPR specification for the Yeast3 network. This expression way is also adopted in other tables of this paper. As the AUPR specification of both the 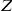-score based method and the method suggested in this paper is better than the best team for the Yeast3 network of DREAM3, score comparisons among them are currently impossible and therefore the scores are omitted.

From these computation results, it is clear that although there are some performance differences among 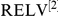, 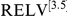, 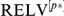 and 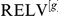, they all show some accuracy improvements in GRN topology inference over the 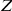-score based method for most of the subchallenges. Especially, significant performance improvements over the best team of both DREAM3 and DREAM4 can even be seen with the estimation method using the 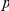-norm based normalization. Moreover, improvements on the AUPR specification are more significant than the AUROC specification. On the other hand, it can be seen that when 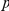 is fixed to be 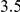, the performance is very close to that of 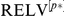 which utilizes the optimal 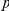. But it is worthwhile to emphasize that in actual applications, there are still no methods for estimating the optimal value of the parameter 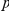, which means that estimation performance comparisons with 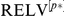 are of little practical values. The purpose of providing results corresponding to the optimal 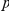 in these tables are only to make it clear that deviations of this parameter from its optimal value generally does not result in significant estimation performance deteriorations.

Results of Tables 1 and 2 also reveal that normalization indeed plays an important role in improving prediction accuracy. As the optimal parameter 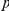 for the 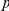-norm based normalization usually can not be known in actual applications, discussions are concentrated on the results of 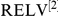, 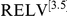 and 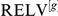. The obtained results show that among these three methods, although the 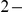
norm based normalization is widely utilized in fields like system analysis and synthesis, signal processing, etc., it seems more appropriate to use the 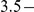
norm based normalization in GRN topology estimations. With this normalization, the suggested algorithm outperforms the 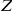-score based method almost in each adopted specification and in every network inference. Improvements in the AUPR specification are particularly evident with the Net1 network and the Net5 network of DREAM4, which are respectively greater than 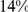 and 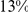. On the other hand, although 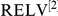 does not perform as well as 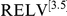, it still yields better results than the 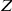-score based method for all the DREAM3 and DREAM4 subchallenges. These facts show that compared with AELV, RELV is indeed more effective in distinguishing direct and indirect regulations, and therefore reducing the so-called cascade errors in GRN topology inference.

In addition, compared with the best team of DREAM3, although the AUROC specification has become slightly worse for some networks, both 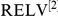 and 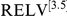 show improvement in the AUPR specification for every network, and the biggest improvement is greater than 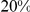. In comparison with the best team of DREAM4, although these two methods occasionally show some great performance decrements, for example, the AUPR specification of 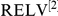 for the Net5 network is about 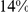 lower than that of the best team, they still yield better results on average in both AUROC and AUPR specifications. According to the report in the web site of the DREAM project for the Size 100 subchallenges of DREAM3, the 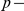
value of the AUPR specification corresponding to the Yeast3 network of the best team is very close to 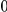 and its score can not be calculated due to some precision difficulties [16]. This has been confirmed by the results reported in Table 1, in which several computed 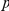-values are zero that is impossible in practice. As the AUPR specifications of 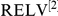, 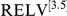, 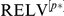 and 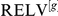 with that network are all higher than that of the best team, the corresponding scores of these estimators can not be computed, either. For the DREAM4 subchallenges, based on the evaluation scripts provided by the DREAM project organizers, the score of the suggested method is computed for every adopted normalization which is also included in Table 2. These results make it clear that both 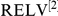 and 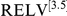 could have ranked the first place in the Size 100 subchallenges of DREAM3 and DREAM4. But it should be emphasized that these comparisons are only of some reference values, noting that all the participants of the DREAM project were completely blind to both the structure and the dynamics of the networks.

Concerning the subchallenges of DREAM4, note that the best team integrated their down ranking method with the 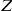-score based method, and the score improvement does not exceed 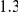 point. On the other hand, the scores of the methods 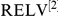 and 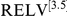 are respectively greater than this best team approximately 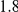 points and 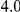 points. These performance improvements appear not to be a small one. When the average ratio is considered for the subchallenges of DREAM3 about the improvements on the AUROC and the AUPR specifications, similar conclusions can also be achieved.

When 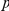-values are directly used in comparing performances of these estimation algorithms, consistent conclusions can be achieved. For example, when the 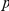-value of the AUPR specification is taken into account for the Net1 network of DREAM4, the values of the 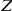-score based method and the best team are respectively about 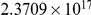 times and 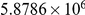 times of that of the method 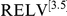.

It appears also worthwhile to note that the best team of DREAM4 utilized an estimation method different from that adopted by the best team of DREAM3. The results of Tables 1 and 2 may imply that the method suggested in this paper shares advantages of different approaches, and overcomes to some extent their disadvantages. But a theoretically solid justification for this declaration is still under investigation, and further efforts are required to clarify the actual reasons behind these phenomena.

Note that in the estimation algorithm suggested in this paper, the step of estimating genes with a zero in-degree plays an important role. To see the effectiveness of the proposed method in this estimation, the number of genes estimated to be of a zero in-degree is given in Table 3 for each network of DREAM3 and DREAM4, together with its actual value. In this estimation, 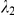, 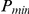 and 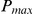 are selected as the same as those adopted in obtaining the estimation results reported in Tables 1 and 2. In this table, an estimation error has also been given which stands for the number of genes that can be regulated by other genes but are estimated to be with a zero in-degree, which is called in this paper, with a slight abuse of terminology, also as a FN (false negative) error.

**Table 3:** Estimated Number of Genes with a Zero In-degree.

	DREAM3					DREAM4				
	Ecoli1	Ecoli2	Yeast1	Yeast2	Yeast3	Net1	Net2	Net3	Net4	Net5
Estimated	15	11	15	8	10	14	6	12	11	7
Actual	17	11	15	8	8	14	10	9	9	8
FN Error	1	0	0	0	0	1	0	0	0	0

Table 3 shows that the suggested method is really effective in estimating genes that can not be regulated by other genes. More detailed analyzes on the estimation results show that if an FN error occurs, then, the genes that are wrongly estimated to be of a zero in-degree are usually regulated by less than 2 other genes. Moreover, if a gene with a zero in-degree is wrongly estimated to be regulated by other genes, then, in the corresponding probabilities, say, 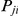
s, the number of values that are significantly greater than 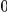 is usually less than 2. These types of mistakes appear reasonable in GRN topology estimation, noting that a large scale GRN usually has a sparse structure and measurement errors may happen to make the estimated value for every RELV of a gene with a small in-degree indistinguishable from 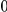. On the contrary, measurement errors are also able to make a few estimated RELVs of a gene with a zero in-degree significantly different from 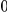.

### Robustness of the Suggested Method

Recall that in the suggested GRN topology estimation algorithm, parameters 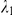, 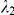, 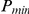 and 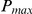 should be selected. While these parameters have some biological interpretations, their selection has not been completely settled from a theoretical viewpoint. It is therefore interesting to investigate how sensitive the estimation accuracy is to the variation of these parameters. As knockout experimental data is used, it appears reasonable to select 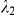 as 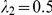. On the other hand, 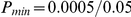 and 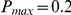 also seem to be an appropriate choice, as a big relative change with a small 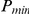 does not result in a significantly large 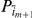, and a great 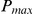 may lead to a large amount of mistakes of wrongly estimating a gene regulated by other genes as a gene with a zero in-degree. These arguments imply that in GRN topology estimation, selection of the parameter 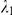 is more essential.

**Figure 1:**
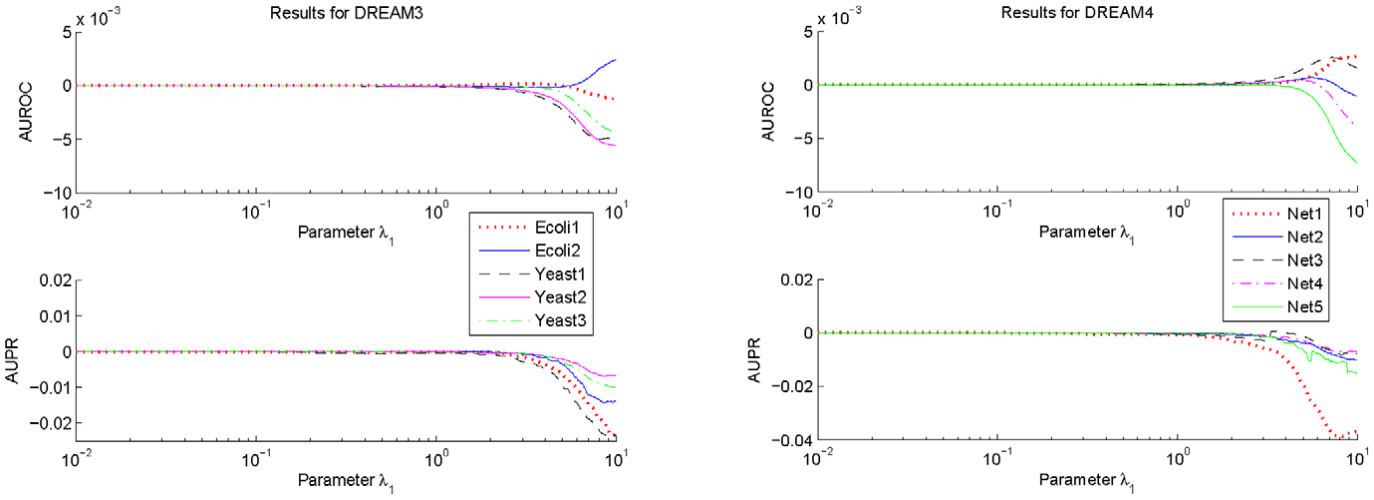
Variations of the AUROC and AUPR specifications of 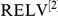 as a function of the parameter 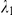. To make the variations clearer, this figure only shows the deviations of the AUROC and the AUPR specifications with the sampled 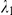 from those with 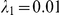.

**Figure 2:**
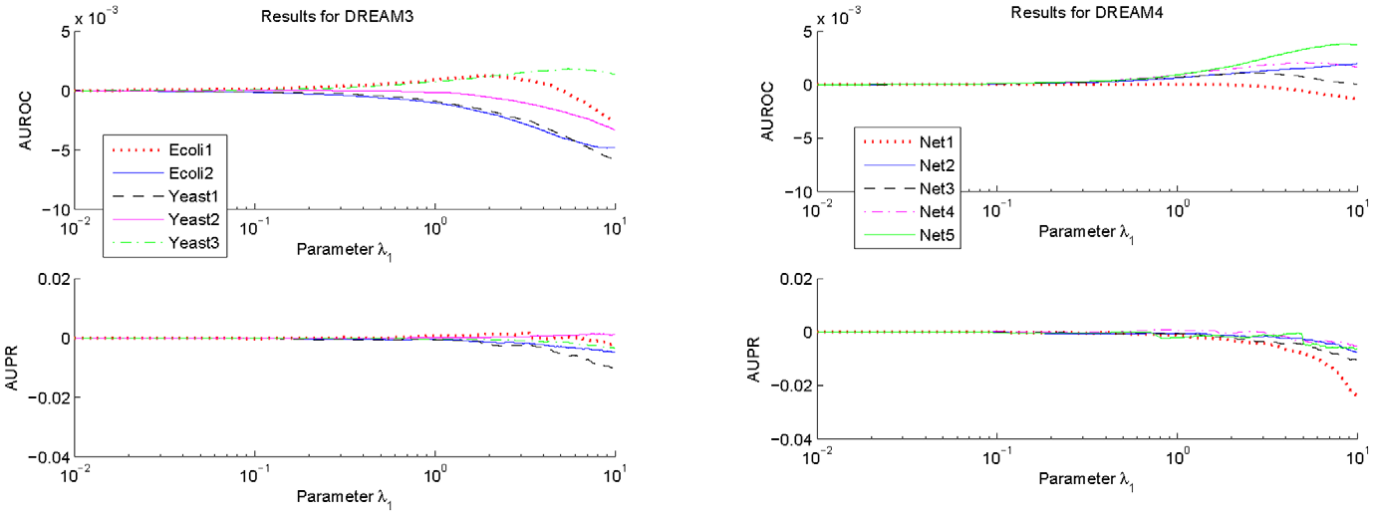
Variations of the AUROC and AUPR specifications of 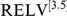 as a function of the parameter 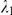. To make the variations clearer, this figure only shows the deviations of the AUROC and the AUPR specifications with the sampled 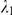 from those with 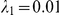.

To investigate influences of the parameter 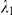 on the prediction accuracy of GRN topology inference, 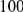 samples are taken for this parameter which is logarithmically equally spaced over the interval 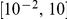. For every sampled parameter 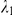, values of AUROC and AUPR for each network of DREAM3 and DREAM4 are calculated with the suggested estimation algorithm using respectively the 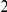-norm and the 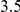-norm based normalizations. The difference between the obtained AUROC specification and that with 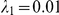, as well as the difference between the obtained AUPR specification and that with 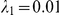, are shown in Figures 1 and 2. In these calculations, 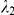, 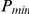 and 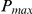 are respectively fixed to be the same values as those used before.

From Figures 1 and 2, it can be seen that performances of the proposed algorithm do vary with the parameter 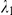. But these performances keep almost the same values if 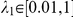. Moreover, except a few networks, these performances begin to decrease from 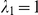. Consistent observations have also been found for the suggested inference algorithm with other 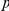-norm based and the geometric average based normalizations. These results imply that in practical applications, it may not be very difficult to select an appropriate 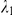. In this paper, this parameter is usually chosen as 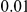.

**Figure 3:**
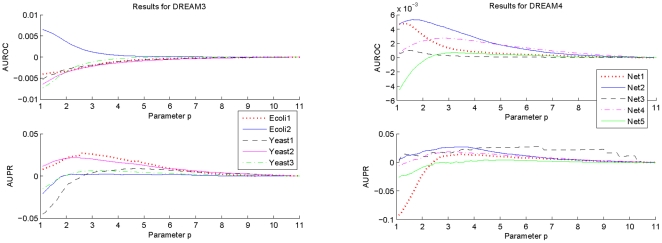
Variations of the AUROC and AUPR specifications of 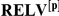 with the increment of the parameter 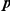. The results shown in this figure are as follows. Ecoli1: AUROC-0.9276, AUPR-0.7019; Ecoli2: AUROC-0.9625, AUPR-0.8665; Yeast1: AUROC-0.9026, AUPR-0.6135; Yeast2: AUROC-0.8601, AUPR-0.4950; Yeast3: AUROC-0.8006, AUPR-0.4561; Net1: AUROC-0.9137, AUPR-0.5494; Net2: AUROC-0.8089, AUPR-0.3824; Net3: AUROC-0.8265, AUPR-0.3917; Net4: AUROC-0.8474, AUPR-0.3719; Net5: AUROC-0.7705, AUPR-0.1921. 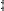 For the Net3 network of DREAM4, the variation interval of the parameter 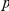 is 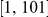.

To understand influences of different normalizations on GRN topology estimation accuracy, variations of the AUROC and AUPR specifications with the parameter 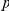 have also been investigated. The results are given in Figure 3. Note that in this figure, the parameter 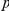 for the Net3 network of DREAM4 should be modified. Its variation interval for this network is 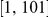. Once again, to make the variations clearer, some particular values have been extracted from the calculated AUROC and AUPR specifications, which are given in detail in the caption of the figure. In these calculations, the parameters 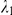, 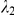, 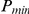 and 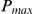 are chosen as the same as those adopted before.

From Figure 3, it is clear that the adopted estimation accuracy metrics indeed vary with the parameter 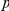. The optimal 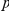 that maximizes the AUROC specification is different from that maximizes the AUPR specification, and different network has a different optimal 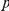. On the other hand, it is also clear from this figure that although the optimal 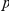 is different for each network and each specification, significant specification change does not arise when the parameter 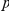 varies over a relatively large interval. More specifically, for each network, the variation of the AUROC specification is not larger than 0.01 in magnitude, and when 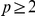, the variation of the AUPR specification is not larger than 0.03 in magnitude. For some particular networks, such as Ecoli2, Yeast3 and Net4, the variation magnitude is much smaller. These observations suggest that in actual applications, it is not very difficult to find a suboptimal value for the parameter 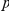. Particularly, 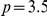 appears to be an appropriate selection for every network of DREAM3 and DREAM4. This can also be confirmed from the results of Tables 1 and 2, which show that, compared with the results with the optimal 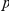, significant performance degradation generally does not arise with the method 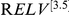. It is worthwhile to note that 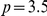 is different from those that are widely adopted in system analysis and synthesis, in which 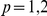, or 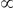 is used more extensively [25], [27].

**Figure 4:**
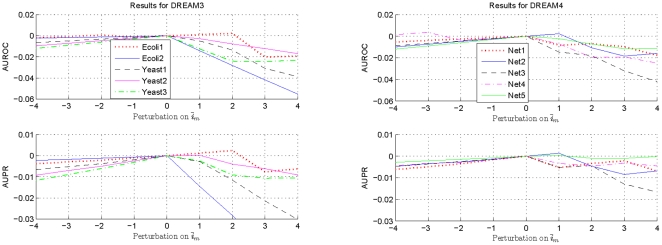
Variations of the AUROC and AUPR specifications of 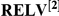 with perturbations on 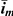. To make the variations clearer, only deviations of the AUROC and AUPR specifications from those of the unperturbed 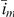 are shown here.

**Figure 5:**
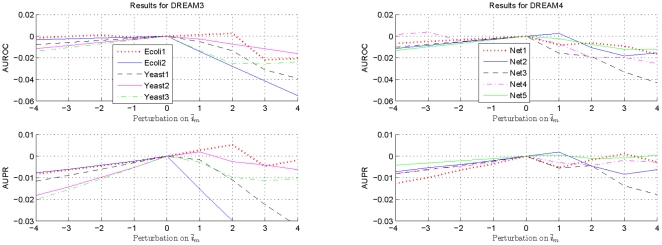
Variations of the AUROC and AUPR specifications of 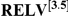 with perturbations on 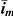. To make the variations clearer, only deviations of the AUROC and AUPR specifications from those of the unperturbed 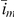 are shown here.

On the other hand, to investigate the validity of the suggested technique for estimating genes with a zero in-degree, the obtained 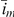 is perturbed to be 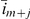 with 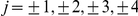. This may simulate the situation under which 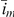 is different from its actual value due to estimation errors in 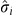 and 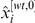, as well as the imperfectness of the adopted assumptions and numerical integration errors, etc. Through the aforementioned perturbations, the estimated number of genes with a zero in-degree can be changed respectively by 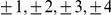 with respect to that of the unperturbed one. The obtained results for the methods 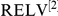 and 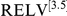 are respectively shown in Figures 4 and 5. When other normalizations are utilized, consistent observations have been obtained and the conclusions are similar. To make the variations clearer, once again, only difference is shown between the obtained specifications and those with the estimated 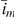.

From Figures 4 and 5, it can be seen that estimation performances with some networks can become slightly better when 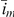 deviates from the value adopted in the suggested estimation algorithm. For example, both the AUROC and the AUPR specifications of the Ecoli1 network and the Net2 network are better when the gene numbered 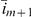 is also regarded to be of a zero in-degree, and the AUPR specification of the Yeast2 network and the Net5 network is a little higher when the gene numbered 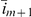 is also considered as a gene not regulated by other genes. However, these performance improvements are not very significant, and when all the networks are taken into account, it is still better to use 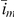 in GRN topology estimations. In addition, if there are small variations in 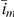, significant performance decrement usually does not arise.

### Performances for Integration of Knockdown and Knockout Data

In this subsection, the suggested method for integrating knockout and knockdown experimental data is applied to the Size 100 subchallenges of both DREAM3 and DREAM 4. As mentioned before, rather than to develop a high performance integration method, the major purposes to include these results are to clarify effectiveness differences of knockout and knockdown experimental data in GRN topology estimations when the suggested method is adopted. In order to compare estimation performances, results using the 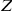-score based method are also integrated with completely the same procedure, that are respectively obtained from the knockout and knockdown experimental data.

The computational results of the Size 100 subchallenges of DREAM3 and DREAM4 are given respectively in Tables 4 and 5, in which 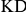, 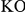 and 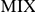 stand respectively for the estimation results obtained from knockdown experimental data only, knockout experimental data only and both of them using the above integration algorithm. Due to space considerations, the reported results are restricted to those with respectively the 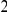-norm and 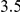-norm based normalization. When other normalizations are utilized, consistent observations have been obtained and the conclusions are similar. For comparisons, the results are also included that are obtained using the 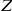-score based method.

**Table 4:** Prediction Performances for the DREAM3 Networks Integrating Knockdown and Knockout Data.

				Ecoli1	Ecoli2	Yeast1	Yeast2	Yeast3	Score
AUROC	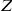 -Score	KD	Area	0.6322	0.6234	0.6606	0.5949	0.5520	
			p-value	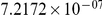	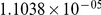	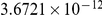	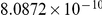	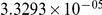	
		KO	Area	0.9131	0.9633	0.8869	0.8470	0.7882	
			p-value	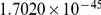	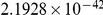	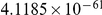	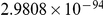	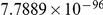	
		MIX	Area	0.9131	0.9631	0.8869	0.8469	0.7881	
			p-value	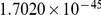	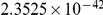	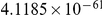	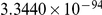	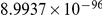	
	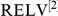	KD	Area	0.6323	0.6457	0.7020	0.6227	0.5717	
			p-value	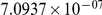	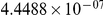	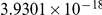	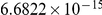	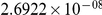	
		KO	Area	0.9243	0.9662	0.8997	0.8565	0.7971	
			p-value	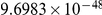	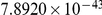	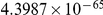	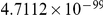	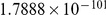	
		MIX	Area	0.9235	0.9654	0.8995	0.8561	0.7969	
			p-value	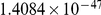	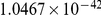	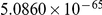	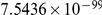	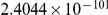	
	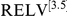	KD	Area	0.6342	0.6479	0.7050	0.6263	0.5738	
			p-value	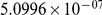	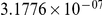	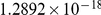	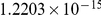	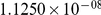	
		KO	Area	0.9262	0.9632	0.9011	0.8584	0.7999	
			p-value	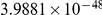	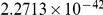	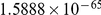	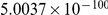	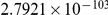	
		MIX	Area	0.9253	0.9624	0.9012	0.8581	0.7997	
			p-value	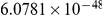	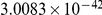	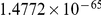	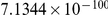	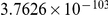	
AUPR	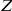 -Score	KD	Area	0.1149	0.1398	0.1088	0.1017	0.1013	15.5823
			p-value	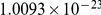	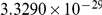	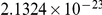	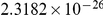	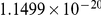	
		KO	Area	0.6919	0.8536	0.5758	0.5076	0.4447	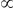
			p-value	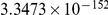	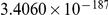	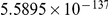	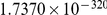	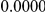 *	
		MIX	Area	0.6687	0.8390	0.5609	0.4847	0.4399	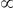
			p-value	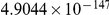	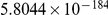	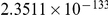	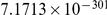	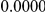 *	
	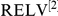	KD	Area	0.1229	0.1599	0.1202	0.1051	0.1071	18.5560
			p-value	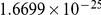	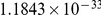	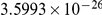	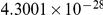	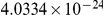	
		KO	Area	0.7230	0.8674	0.6013	0.5163	0.4569	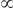
			p-value	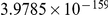	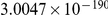	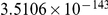	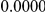 *	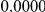 *	
		MIX	Area	0.6224	0.7814	0.5389	0.4731	0.4398	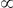
			p-value	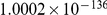	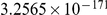	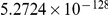	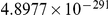	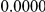 *	
	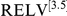	KD	Area	0.1180	0.1603	0.1214	0.1055	0.1071	18.6934
			p-value	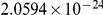	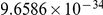	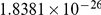	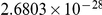	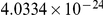	
		KO	Area	0.7260	0.8686	0.6191	0.5130	0.4625	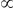
			p-value	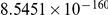	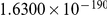	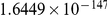	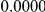 *	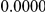 *	
		MIX	Area	0.6122	0.7721	0.5357	0.4662	0.4401	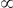
			p-value	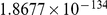	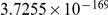	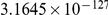	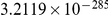	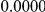 *	

KD: estimation performance using knockdown experimental data only; KO: estimation performance using knockout experimental data only; MIX: estimation performance using both knockdown and knockout experimental data.

*Due to the some reasons as those of Table 1, these 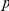-values can not be distinguished from zero in actual computations, which makes it impossible to compare scores of the corresponding GRN topology estimation methods. Using the same treatments of [16], these scores are designated to be 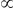.

**Table 5:** Prediction Performances for the DREAM4 Networks Integrating Knockdown and Knockout Data.

				Net1	Net2	Net3	Net4	Net5	Score
AUROC	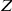 -Score	KD	Area	0.7582	0.6923	0.6414	0.7348	0.6600	
			p-value	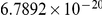	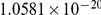	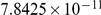	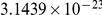	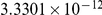	
		KO	Area	0.9132	0.8015	0.8328	0.8424	0.7583	
			p-value	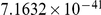	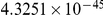	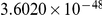	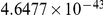	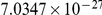	
		MIX	Area	0.9143	0.8022	0.8330	0.8428	0.7594	
			p-value	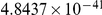	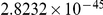	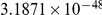	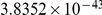	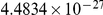	
	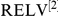	KD	Area	0.7568	0.6649	0.6456	0.7271	0.6615	
			p-value	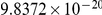	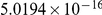	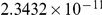	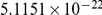	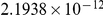	
		KO	Area	0.9168	0.8141	0.8271	0.8498	0.7699	
			p-value	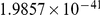	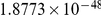	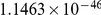	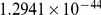	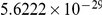	
		MIX	Area	0.9178	0.8149	0.8272	0.8502	0.7711	
			p-value	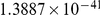	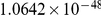	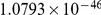	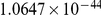	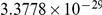	
	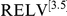	KD	Area	0.7559	0.6676	0.6482	0.7315	0.6531	
			p-value	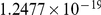	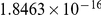	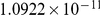	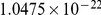	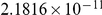	
		KO	Area	0.9147	0.8123	0.8274	0.8500	0.7711	
			p-value	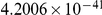	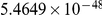	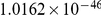	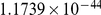	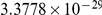	
		MIX	Area	0.9154	0.8144	0.8277	0.8510	0.7725	
			p-value	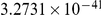	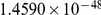	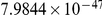	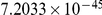	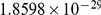	
AUPR	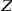 -Score	KD	Area	0.3175	0.1758	0.1224	0.2059	0.0716	30.4375
			p-value	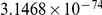	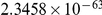	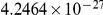	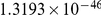	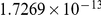	
		KO	Area	0.4927	0.3881	0.3814	0.3685	0.1703	70.3408
			p-value	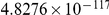	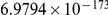	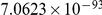	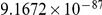	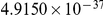	
		MIX	Area	0.5851	0.4224	0.4054	0.4089	0.2195	77.3406
			p-value	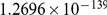	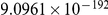	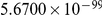	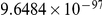	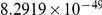	
	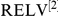	KD	Area	0.3278	0.1712	0.1227	0.1990	0.0710	29.7591
			p-value	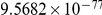	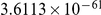	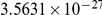	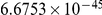	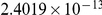	
		KO	Area	0.5274	0.4011	0.3935	0.3806	0.1836	73.4399
			p-value	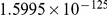	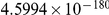	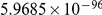	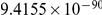	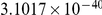	
		MIX	Area	0.5989	0.4280	0.4058	0.4193	0.2283	79.0832
			p-value	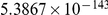	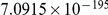	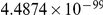	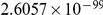	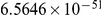	
	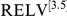	KD	Area	0.3304	0.1710	0.1254	0.1927	0.0666	29.6565
			p-value	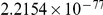	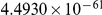	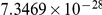	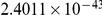	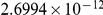	
		KO	Area	0.5638	0.4100	0.4061	0.3901	0.1928	75.5441
			p-value	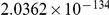	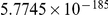	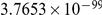	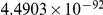	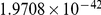	
		MIX	Area	0.6030	0.4284	0.4050	0.4247	0.2308	79.3772
			p-value	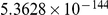	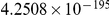	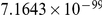	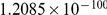	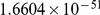	

KD: estimation performance using knockdown experimental data only; KO: estimation performance using knockout experimental data only; MIX: estimation performance using both knockdown and knockout experimental data.

From Tables 4 and 5, it is clear that when applied to the DREAM4 subchallenges, the suggested integration procedure is able to improve estimation performances for both the 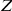-score based method and the estimation algorithm suggested in this paper. As a matter of fact, compared with the results using only knockout experimental data, although there is one network with which the AUPR specification has been slightly degraded when the method 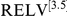 is used, the final score of 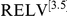 has been increased by about 3.8 points. Furthermore, the scores of the method 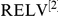 and the Z-score based method have been increased more significantly, which are respectively about 5.6 and 7.0 points. These improvements seem not small, noting that the best team of DREAM4 integrated the 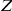-score based method with their down ranking method, but the obtained merits are less than 1.3 points. In addition, this integration method appears more effective for the 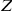-score based method. More specifically, under such a situation, for each network, every adopted specification has been improved, and the final score has been increased almost 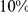. These observations are significantly different from those of [18]–[20], which indicated that when knockout experimental data are available, knockdown experimental data is of little values in GRN topology inference.

Similar conclusions can be achieved if the 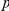-values of the obtained estimation specifications are directly compared.

However, when utilized in the DREAM3 subchallenges, the aforementioned integration procedure does not work very well either with the 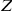-score based estimation algorithm or the algorithm suggested in this paper. Compared with the results using only knockout experimental data, this integration even worsen almost every specification of each network. The reasons are still not clear which are worthy of further efforts. But from these observations, it is clear that compared with those of DREAM3, information in the data sets of DREAM4 about the structure of a GRN are more consistent which are respectively contained in the knockout and knockdown experimental data.

Note that although in DREAM3, only measurement errors are added into the simulated experimental data, variances of the measurement errors are assumed to be of the same value for every gene under all situations, no matter it is in the wild type, or when some genes of the GRN have been knocked out or knocked down. On the other hand, in DREAM4, external disturbances are added to both the simulated mRNA concentrations and the simulated protein concentrations, but both background noises and the fact that gene expression values are typically measured on a logarithmic scale have been taken into account in simulating these external disturbances. Such a treatment makes a simulated measurement error have a standard variance approximately proportional to a simulated actual value of the expression level of a gene [16], [17]. Note that the magnitude of a knockout perturbation is twice as that of a knockdown perturbation. It can therefore be declared that compared with the knockdown experimental data of DREAM4, those of DREAM3 are more noisy, and hence less informative. These can also be seen from the differences between the AUROC/AUPR specifications using respectively only the knockout experimental data and the knockdown experimental data. As a matter of fact, it is clear from Tables 4 and 5 that for all the adopted estimation methods, compared with their counterparts in network topology estimations of DREAM4, the above differences are consistently larger in those of DREAM3, especially when the AUPR specification is considered. As the simulated data of DREAM4 are believed to be closer to actual biological experimental data than those of DREAM3 [14], [17], [20], it is hoped that the suggested integration method is helpful in practical GRN topology estimations.

In addition to these, it is also clear from these tables that when only knockdown experimental data is utilized, the 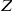-score based method outperforms about 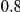 point the method suggested in this paper with the DREAM4 subchallenges. But when the DREAM3 subchallenges are coped with, the conclusions are completely the opposite, in which the methods suggested in this paper, no matter the method 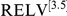 or the method 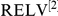, can obtain a score higher than the 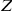-score based method approximately 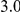 points.

### Performances for Integration with the Down Ranking Algorithm

In this subsection, estimations are performed using the integration procedure proposed for the suggested RELV based inference method and the so called down ranking method. The corresponding results are given in Tables 6 and 7 when the 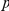-norm based normalization with 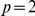 and 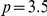 are respectively used in these method integrations. The corresponding results are given in the rows started by 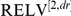 and 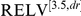 respectively. To compare the effectiveness of method integration, results obtained through integrating the 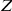-score based method and the down ranking method are also included, which are denoted by 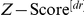. In these tables, only results with some typical and optimal values for the threshold of the down ranking method are included. In searching the optimal threshold value, the AUPR specification is once again taken as the cost function.

**Table 6:** Prediction Performances for the DREAM3 Networks Using Method Integrations.†

			Ecoli1	Ecoli2	Yeast1	Yeast2	Yeast3
AUROC	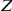 -Score	Area	0.9131	0.9633	0.8869	0.8470	0.7882
		p-value	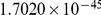	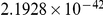	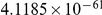	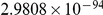	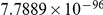
	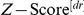	Area	0.9133	0.9636	0.8875	0.8480	0.7887
	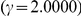	p-value	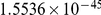	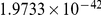	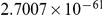	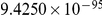	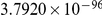
	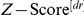	Area	*0.9133*	*0.9637*	*0.8873*	*0.8477*	*0.7887*
	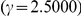	p-value	*1.5536* 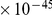	*1.9051* 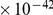	*3.1089* 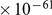	*1.3318* 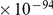	*3.7920* 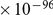
	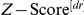	Area	*0.9134*	*0.9637*	*0.8875*	*0.8478*	*0.7887*
	(optimal 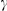 )	p-value	*1.4843* 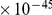	*1.9051* 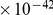	*2.7007* 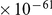	*1.1868* 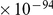	*3.7920* 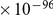
		opt. 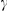	(2.1000)	(2.2000)	(2.0000)	(2.2500)	(2.5000)
	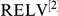	Area	0.9243	0.9662	0.8997	0.8565	0.7971
		p-value	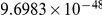	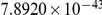	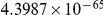	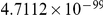	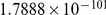
	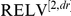	Area	0.9245	0.9665	0.9001	0.8570	0.7975
	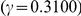	p-value	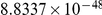	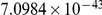	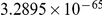	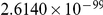	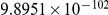
	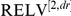	Area	*0.9245*	*0.9665*	*0.9001*	*0.8571*	*0.7976*
	(optimal 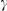 )	p-value	*8.8337* 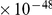	*7.0984* 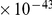	*3.2895* 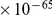	*2.3232* 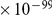	*8.5327* 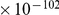
		opt. 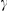	(0.3200)	(0.3100)	(0.3000)	(0.3200)	(0.2900)
	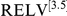	Area	0.9262	0.9632	0.9011	0.8584	0.7999
		p-value	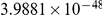	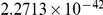	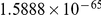	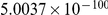	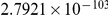
	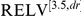	Area	0.9263	0.9634	0.9013	0.8587	0.7999
	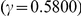	p-value	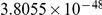	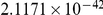	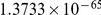	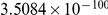	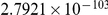
	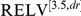	Area	*0.9263*	*0.9634*	*0.9014*	*0.8587*	*0.8000*
	(optimal 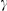 )	p-value	*3.8055* 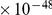	*2.1171* 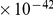	*1.2768* 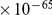	*3.5084* 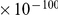	*2.4050* 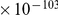
		opt. 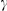	(0.6600)	(0.5200)	(0.6200)	(0.5800)	(0.6500)
AUPR	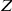 -Score	Area	0.6919	0.8536	0.5758	0.5076	0.4447
		p-value	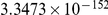	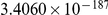	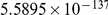	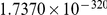	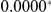
	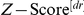	Area	0.7295	0.8899	0.6521	0.5609	0.4799
	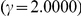	p-value	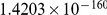	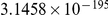	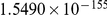	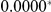	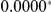
	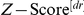	Area	*0.7288*	*0.8947*	*0.6336*	*0.5590*	*0.4857*
	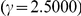	p-value	*2.0335* 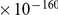	*2.7247* 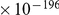	*4.8925* 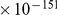	*0.0000* *	*0.0000* *
	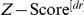	Area	*0.7307*	*0.8966*	*0.6521*	*0.5663*	*0.4857*
	(optimal 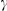 )	p-value	*7.6767* 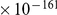	*1.0346* 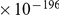	*1.5490* 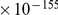	*0.0000* *	*0.0000* *
		opt. 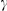	(2.1000)	(2.2000)	(2.0000)	(2.2500)	(2.5000)
	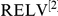	Area	0.7230	0.8674	0.6013	0.5163	0.4569
		p-value	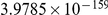	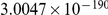	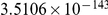	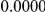 *	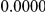 *
	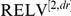	Area	0.7487	0.8961	0.6440	0.5517	0.4857
	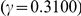	p-value	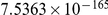	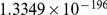	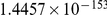	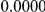 *	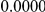 *
	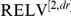	Area	*0.7496*	*0.8961*	*0.6453*	*0.5544*	*0.4903*
	(optimal 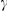 )	p-value	*4.7507* 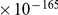	*1.3349* 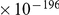	*6.9809* 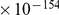	*0.0000* *	*0.0000* *
		opt. 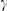	(0.3200)	(0.3100)	(0.3000)	(0.3200)	(0.2900)
	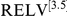	Area	0.7260	0.8686	0.6191	0.5130	0.4625
		p-value	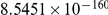	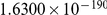	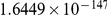	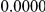 *	0.0000*
	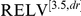	Area	0.7398	0.8845	0.6382	0.5350	0.4615
	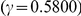	p-value	7.2260 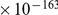	4.9311 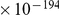	3.7217 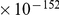	0.0000*	0.0000*
	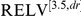	Area	*0.7424*	*0.8882*	*0.6435*	*0.5350*	*0.4696*
	(optimal 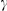 )	p-value	*1.9053* 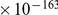	*7.4817* 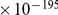	*1.9129* 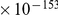	*0.0000* *	*0.0000* *
		opt. 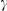	(0.6600)	(0.5200)	(0.6200)	(0.5800)	(0.6500)

† As noted in [22], 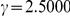 is obtained for 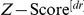 after a comparison with the actual network. On the other hand, the optimal 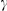 can hardly be obtained in actual estimations for each of 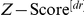, 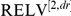, 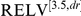. The purposes to include their inference results here are only to clarify estimation performance degradations when an empirical parameter 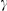 is adopted.

*Due to the some reasons as those of Table 1, these 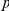-values can not be distinguished from zero in actual computations, which makes it impossible to compare scores of the adopted GRN topology estimation methods.

**Table 7:** Prediction Performances for the DREAM4 Networks Using Method Integrations.†

			Net1	Net2	Net3	Net4	Net5	Score
AUROC	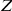 -Score	Area	0.9132	0.8015	0.8328	0.8424	0.7583	
		p-value	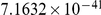	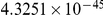	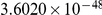	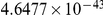	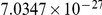	
	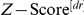	Area	0.9133	0.8014	0.8329	0.8418	0.7592	
	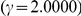	p-value	6.9131 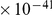	4.5965 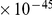	3.3882 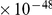	6.1986 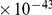	4.8666 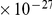	
	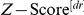	Area	*0.9150*	*0.8018*	*0.8331*	*0.8432*	*0.7570*	
	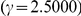	p-value	*3.7747* 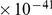	*3.6028* 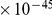	*2.9978* 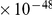	*3.1641* 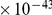	*1.1956* 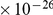	
	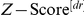	Area	*0.9150*	*0.8018*	*0.8332*	*0.8433*	*0.7595*	
	(optimal 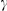 )	p-value	*3.7747* 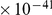	*3.6028* 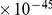	*2.8197* 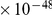	*3.0155* 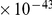	*4.3031* 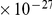	
		opt. 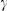	(2.5000)	(2.5500)	(2.4500)	(2.4500)	(2.1000)	
	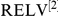	Area	0.9168	0.8141	0.8271	0.8498	0.7699	
		p-value	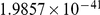	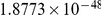	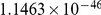	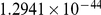	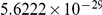	
	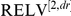	Area	0.9180	0.8142	0.8274	0.8504	0.7704	
	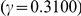	p-value	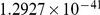	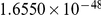	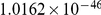	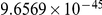	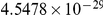	
	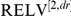	Area	*0.9182*	*0.8143*	*0.8274*	*0.8504*	*0.7707*	
	(optimal 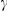 )	p-value	*1.2034* 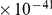	*1.5539* 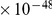	*1.0162* 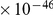	*9.6569* 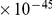	*4.0039* 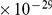	
		opt. 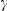	(0.2700)	(0.3400)	(0.3100)	(0.3100)	(0.2400)	
	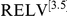	Area	0.9147	0.8123	0.8274	0.8500	0.7711	
		p-value	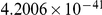	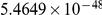	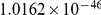	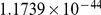	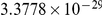	
	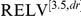	Area	0.9155	0.8124	0.8274	0.8504	0.7713	
	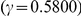	p-value	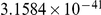	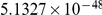	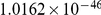	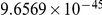	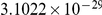	
	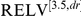	Area	*0.9155*	*0.8124*	*0.8275*	*0.8504*	*0.7717*	
	(optimal 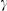 )	p-value	*3.1584* 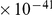	*5.1327* 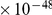	*9.0080* 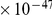	*9.6569* 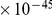	*2.6163* 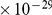	
		opt. 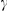	(0.5500)	(0.6000)	(0.6200)	(0.5900)	(0.3700)	
AUPR	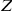 -Score	Area	0.4927	0.3881	0.3814	0.3685	0.1703	70.3408
		p-value	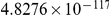	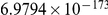	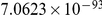	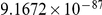	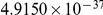	
	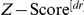	Area	0.5361	0.3771	0.3898	0.3494	0.2133	71.5899
	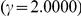	p-value	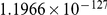	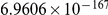	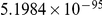	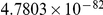	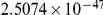	
	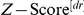	Area	*0.6591*	*0.4144*	*0.4119*	*0.4446*	*0.2000*	*79.2944*
	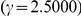	p-value	*1.0473* 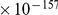	*2.4387* 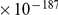	*1.2670* 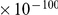	*1.4705* 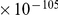	*3.7607* 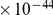	
	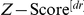	Area	*0.6591*	*0.4143*	*0.4148*	*0.4470*	*0.2141*	
	(optimal 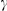 )	p-value	*1.0473* 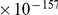	*2.7696* 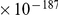	*2.3240* 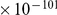	*3.7559* 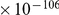	*1.6150* 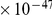	*79.7692*
		opt. 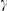	(2.5000)	(2.5500)	(2.4500)	(2.4500)	(2.1000)	
	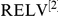	Area	0.5274	0.4011	0.3935	0.3806	0.1836	73.4399
		p-value	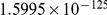	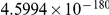	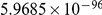	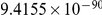	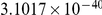	
	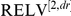	Area	0.6454	0.4191	0.4218	0.4518	0.2040	80.3293
	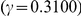	p-value	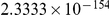	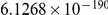	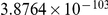	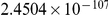	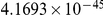	
	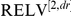	Area	*0.6562*	*0.4209*	*0.4214*	*0.4514*	*0.2230*	
	(optimal 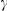 )	p-value	*5.3549* 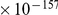	*6.1692* 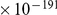	*4.8979* 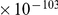	*3.0763* 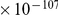	*1.2102* 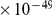	*81.0974*
		opt. 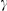	(0.2700)	(0.3400)	(0.3100)	(0.3100)	(0.2400)	
	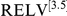	Area	0.5638	0.4100	0.4061	0.3901	0.1928	75.5441
		p-value	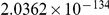	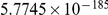	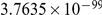	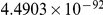	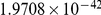	
	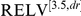	Area	0.6313	0.4149	0.4094	0.4275	0.2024	78.7214
	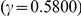	p-value	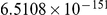	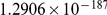	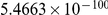	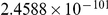	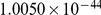	
	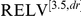	Area	*0.6340*	*0.4170*	*0.4209*	*0.4286*	*0.2242*	
	(optimal 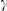 )	p-value	*1.4250* 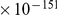	*8.9022* 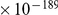	*6.5615* 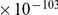	*1.3154* 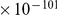	*6.2559* 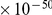	*79.7579*
		opt. 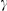	(0.5500)	(0.6000)	(0.6200)	(0.5900)	(0.3700)	

† The purposes to include the inference results of 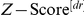 with 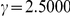, 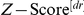 with the optimal 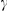, 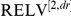 with the optimal 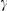, 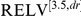 with the optimal 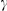, are completely the same as those of Table 6. That is, to clarify estimation performance degradations when an empirical parameter 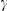 is adopted for these methods.

Similar to Tables 1 and 2, estimation results with the optimal threshold, as well as those of 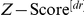 with 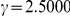, are included here only for some references. The major purposes for this inclusion are to clarify estimation performance degradations when the adopted threshold 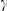 deviates from its optimal value.

From Tables 6 and 7, it is obvious that the down ranking method is indeed helpful in improving estimation accuracy, no matter it is integrated with the 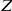-score based method or the method suggested in this paper. Moreover, compared with the AUROC specification, the AUPR specification has been improved more significantly. In addition, estimation performances for the DREAM4 subchallenges have been improved more greatly than those of the DREAM3 subchallenges.

An interesting observation from these tables is that when the optimal threshold value is adopted for the down ranking method, although the method 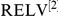 still outperforms the 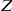-score based method, performance differences among the methods 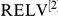, 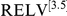 and the 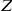-score based method become smaller than those before the method integration. While this may mean that the down ranking method is more effective in improving the 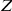-score based method, it may also suggest that the method proposed in this paper is really effective in reducing the so-called cascade errors in GRN topology estimations.

The results of Tables 6 and 7 also indicate that although the algorithm suggested in this paper is able to reduce the so called cascade errors, there still exist some cascade errors that this algorithm fails to detect. This may possibly be due to the following three causes. One is the imperfectness of the experimental data in which several kinds of noises exist. One is the imperfectness of the adopted assumptions on measurement errors, which may have not appropriately reflected their actual distributions. The other one is that there may exist genes for which indirect regulations cause a RELV with a magnitude bigger than that caused by direct regulations.

On the other hand, it seems that the down ranking method is much more helpful in improving the prediction performance of the method 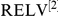 than that of the method 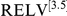.

**Figure 6:**
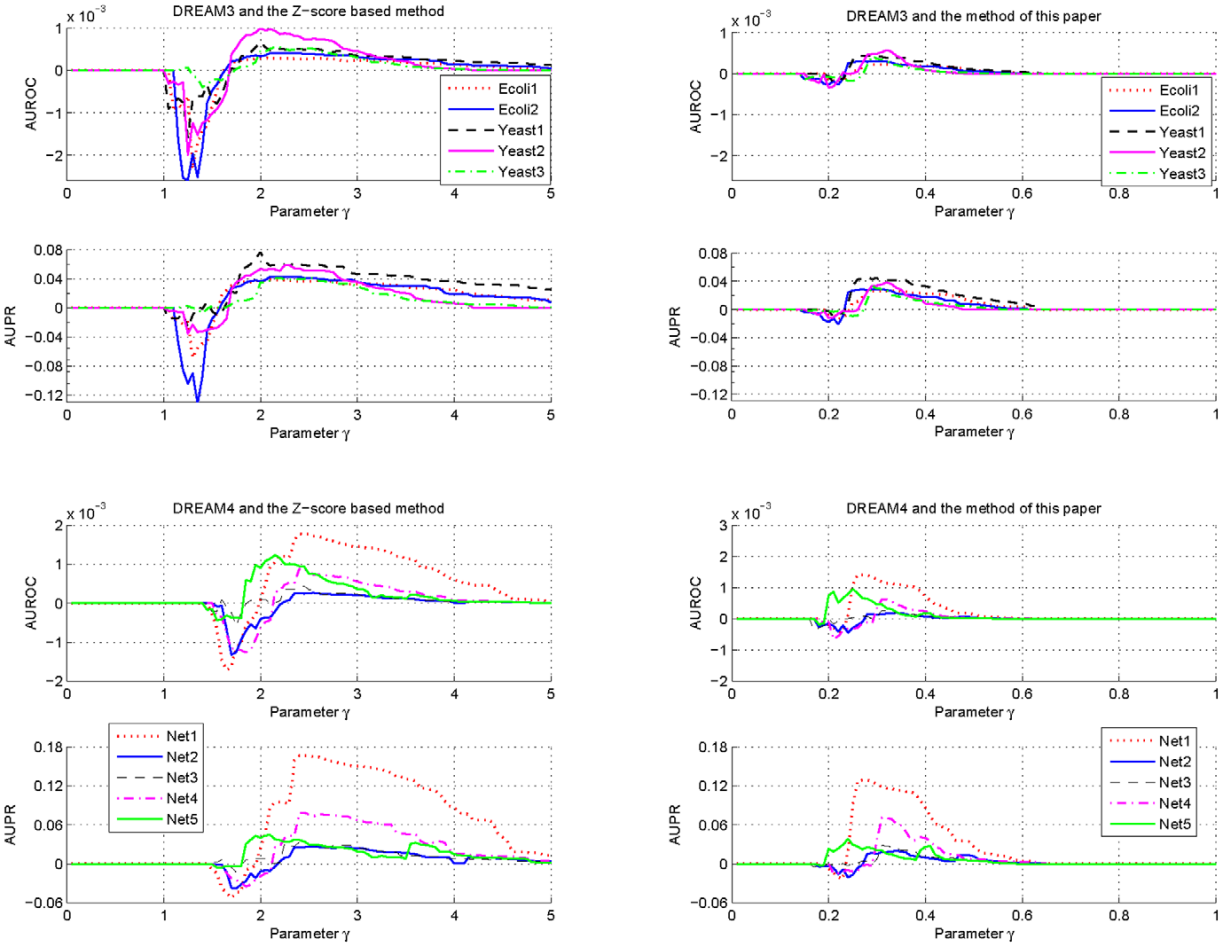
Variations of the AUROC and AUPR specifications with the threshold value 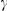
 **.** To make the variations clearer, the specifications shown are their deviations from those respectively with 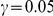 (for the 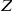-score based method) and with 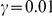 (for the algorithm suggested in this paper).

In applying the down ranking algorithm, a threshold value 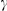 should be chosen for extracting a primary estimation about the network structure from some computed weights or confidences about direct regulations between any two different genes of a GRN. There is, however, still no very solid theoretical guidance on how to suitably choose this threshold value [22]. As an example, it is reported in [22] that while 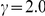 is found through extensive numerical simulations to be the best selection for integrating with the Z-score based method, 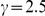 is more appropriate for the subchallenges of DREAM4. It is therefore interesting to investigate variations of estimation performances with this parameter. Due to space considerations and the fact that 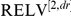 outperforms 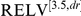, discussions are restricted to the method with the 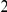-norm based normalization. When the 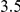-norm based normalization is utilized, consistent observations have been obtained and the conclusions are similar. According to the results reported in [22], when the 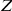-score based method is to be integrated, the interval for the parameter 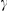 is selected to be 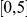 in this paper. On the other hand, when the method suggested in this paper is to be integrated, this interval is chosen as 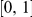. In these intervals, 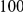 equally spaced samples are used in searching the optimal 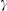. Figure 6 shows these variations when the 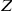-score based method and the algorithm suggested in this paper with the 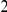-norm based normalization are respectively integrated with the down ranking method.

Variations of the AUROC and the AUPR specifications with the parameter 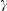 are shown in Figure 6. From this figure, it is clear that although the optimal value is different for each network and each specification, 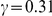 appears to be a good choice for the threshold value when the down ranking method is integrated with the estimation method suggested in this paper. The corresponding results for the Size 100 subchallenges of DREAM3 and DREAM4 are given respectively in Tables 6 and 7, together with those using the optimal 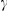.

The results of Figure 6 are also consistent with the observations reported in [22]. That is, when the 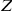-score based method is integrated with the down ranking method, 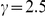 is more appropriate for the Size 100 subchallenges of DREAM4, although 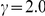 is generally believed to be the best selection.

From Figure 6, it is also clear that compared with the 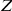-score based method, estimation performances of the algorithm suggested in this paper is less sensitive to variations of the threshold around its optimal value, when they are respectively integrated with the down ranking method. This property is attractive in practical applications, recalling that it is still not very clear how to choose the optimal threshold value for a particular GRN and an experienced value usually deviates from the optimal one.

### Further Discussions

**Figure 7:**
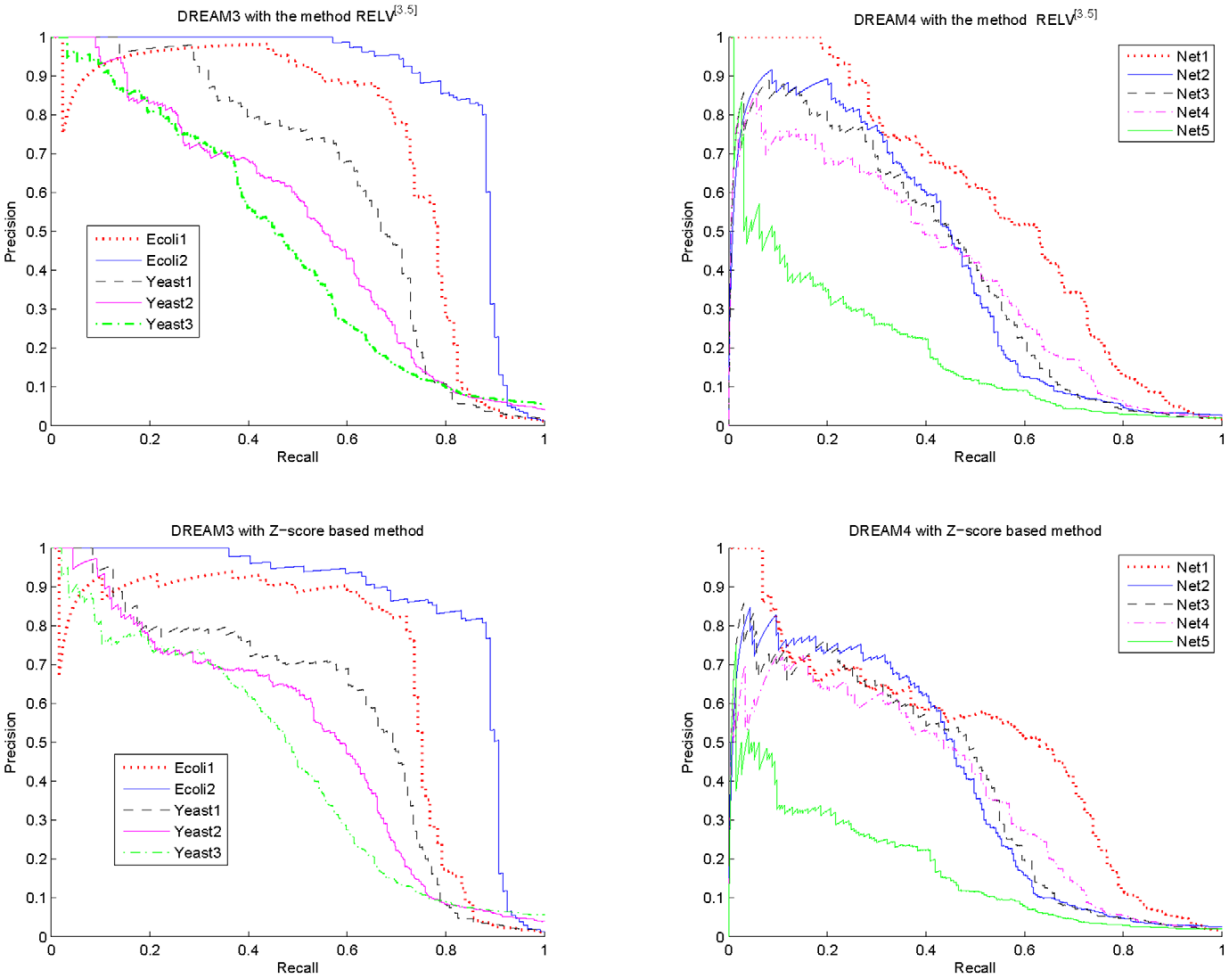
Precision-recall curves of some typical estimations.

As commented in [16], highly confident predictions in GRN topology estimations can become a good guidance to biological experiment designs. However, these predictions will be helpful only if their precisions are also sufficiently high. This requirement asks that a desirable estimation algorithm should have a PR (precision-recall) curve starting from the left upper corner, and decreasing monotonically and slowly with the increment of the recall rate. To see whether predictions made by the algorithm suggested in this paper share this property, the PR curve of the method 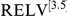 is shown in Figure 7 for each network of the Size 100 subchallenges of DREAM3 and DREAM4, which is based only on the knockout experimental data. To compare satisfaction degree about this requirement with the Z-score based method, its corresponding PR curve for each network is also included.

From this figure, it is obvious that for every network of DREAM3, when the recall rate is around 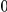, the prediction precision of the suggested estimation method is approximately equal to 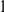, and this prediction precision keeps large when the recall rate is less than some value. Moreover, this value is specially large for the Ecoli2 network. This suggests that for the DREAM3 Size 100 network inference subchallenges, predictions with a high confidence obtained by the suggested method are usually correct and are therefore helpful in the design of a follow-up experiment validation. This is different from the algorithm used by the best team and the second place team of DREAM3, which may not be very desirable in this aspect [16].

However, when applied to the DREAM4 subchallenges, the aforementioned properties do not hold for most of the networks. As a matter of fact, except the Net1 and Net5 networks, the PR curve even does not start from the upper left corner. This means that there still exist some false positive errors among the estimated direct regulations whose existence is predicted with a high confidence by the suggested method. Furthermore, when the suggested method is integrated with the down ranking method, similar observations have been obtained. On the other hand, when the Z-score based method is utilized, consistent phenomena have been observed.

Nevertheless, a detailed analysis shows that concerning this requirement on GRN topology estimators, the 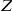-score based method does not outperform the method suggested in this paper, either. As an obvious example, in the DREAM4 subchallenges, when the Z-score based method is utilized, only the Net1 network has a PR curve starting from the upper left corner. More detailed comparisons are omitted, but it can be claimed from Figure 7 that the method suggested in this paper appears more helpful than the Z-score based method in guiding the design of a biological experiment to validate the actual existence of a predicted direct regulation.

When the Z-score based method is integrated with the down ranking method, which is adopted by the best team of DREAM4, the corresponding PR curves for these benchmark networks are very similar to those obtained from the Z-score based method. This implies that further enhancements are still required to make this integration applicable to practical GRN structure inferences.

Computations have been performed also on many other simulated large scale GRNs. The observed phenomena are consistent with what have been reported in this section.

### Concluding Remarks

In this paper, an algorithm is developed for inferring GRN topology from steady state knockout/knockdown experimental data. Rather than the commonly used AELVs (absolute expression level variation), it utilizes RELVs (relative expression level variation) of a gene in gene knockout/knockdown experiments to measure possibilities of the existence of direct regulations among genes. Based on this variation, probability is estimated from experimental data for the existence of a regulation between two different genes of a GRN, which is further used to estimate whether or not a gene is regulated by any other genes. The estimated magnitude of the RELV of a gene is normalized and modified, on the basis of the estimation results about the existence of direct regulations to it. These normalized and modified magnitudes are used in queuing the possibility of the existence of a corresponding direct regulation. A distinguished characteristic of this algorithm is that its computational complexity increases only quadratically with the number of genes in a GRN.

Computational results with the Size 100 subchallenges of both DREAM3 and DREAM4 show that this method can outperform not only the widely used 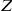-score based method, but also the best team of these subchallenges who used an integration of some well known methods. While these comparisons are only of some reference values, as all the DREAM project participants were completely blinded to both the structure and the dynamics of the simulated networks, it appears safe to claim that the suggested method is more efficient than the available methods in distinguishing direct and indirect regulations of a GRN. Integration with the so-called down ranking method show that the so-called cascade errors in GRN topology estimations can be further reduced. Precision analyzes show that highly confident predictions obtained by this method are usually more helpful in guiding designs of a biological validation experiment than those by the 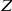-score based method.

Further efforts along this line appear to test effectiveness of the suggested method with actual biological experimental data, to extend the suggested estimation method to biological experimental data in which several genes are simultaneously perturbed by external interferences, to give a more biologically significant normalization of the RELVs and selection of the parameters 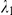, 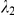, 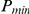 and 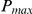, as well as to improve estimation accuracy of gene expression levels in the wild type and that of the variance of measurement errors. Challenges still remains there in reducing false positive errors among highly confident predictions, especially when the RELV of an indirect regulation is larger in magnitude than that of some direct regulations. It is also interesting to see whether some other structure information about a GRN, such as the power law, etc., can be helpful in making a more accurate prediction.
